# Therapeutic effect of psilocybin in addiction: A systematic review

**DOI:** 10.3389/fpsyt.2023.1134454

**Published:** 2023-02-09

**Authors:** Pim B. van der Meer, Juan J. Fuentes, Ad A. Kaptein, Jan W. Schoones, Marleen M. de Waal, Anneke E. Goudriaan, Kees Kramers, Arnt Schellekens, Metten Somers, Matthijs G. Bossong, Albert Batalla

**Affiliations:** ^1^Department of Neurology, Leiden University Medical Center, Leiden, Netherlands; ^2^Department of Psychiatry, University Medical Center Utrecht Brain Center, University Medical Center Utrecht, Utrecht, Netherlands; ^3^Addiction Program, Institute of Neuropsychiatry and Addictions (INAD), Hospital del Mar, Barcelona, Spain; ^4^Addiction Research Group, IMIM Hospital del Mar Medical Research Institute, Barcelona, Spain; ^5^Department of Psychiatry and Legal Medicine, Universitat Autònoma de Barcelona, Cerdanyola del Vallés, Spain; ^6^Department of Medical Psychology, Leiden University Medical Center, Leiden, Netherlands; ^7^Directorate of Research Policy, Leiden University Medical Center, Leiden, Netherlands; ^8^Department of Research and Jellinek, Arkin Mental Health Care, Amsterdam, Netherlands; ^9^Department of Psychiatry, Amsterdam University Medical Center, Amsterdam, Netherlands; ^10^Amsterdam Institute for Addiction Research, Amsterdam University Medical Center, Amsterdam, Netherlands; ^11^Department of Pharmacology-Toxicology, Radboud University Medical Center, Nijmegen, Netherlands; ^12^Nijmegen Institute for Science Practitioners in Addiction (NISPA), Nijmegen, Netherlands; ^13^Department of Psychiatry, Radboud University Medical Center, Nijmegen, Netherlands

**Keywords:** psilocybin, hallucinogens, substance-related disorders, substance abuse, drug addiction, psychotherapy

## Abstract

**Background:**

Psychedelic-assisted therapy [e.g., with lysergic acid diethylamide (LSD)] has shown promising results as treatment for substance use disorders (SUDs). Previous systematic reviews assessing the efficacy of psilocybin in SUDs only included clinical trials conducted in the last 25 years, but they may have missed clinical trials assessing the efficacy of psilocybin that were conducted before the 1980s, given much research has been done with psychedelics in the mid-20th century. In this systematic review, we specifically assessed the efficacy of psilocybin in patients with a SUD or non-substance-related disorder with no publication date restrictions in our search strategy.

**Methods:**

A systematic literature search was performed according to Preferred Reporting Items for Systematic reviews and Meta-Analysis (PRISMA) guidelines from the earliest published manuscript up to September 2, 2022, in seven electronic databases, including clinical trials in patients with a SUD or non-substance-related disorder evaluating the efficacy of psilocybin.

**Results:**

A total of four studies (six articles, of which two articles were long-term follow-up results from the same trial) were included in this systematic review. Psilocybin-assisted therapy was administered to *n* = 151 patients in a dose ranging from 6 to 40 mg. Three studies focused on alcohol use disorder, and one study on tobacco use disorder. In a pilot study (*n* = 10), the percentage of heavy drinking days decreased significantly between baseline and weeks 5–12 (mean difference of 26.0, 95% CI = 8.7–43.2, *p* = 0.008). In another single-arm study (*n* = 31), 32% (10/31) became completely abstinent from alcohol (mean duration of follow-up 6 years). In a double-blind, placebo-controlled randomized controlled trial (RCT, *n* = 95), the percentage of heavy drinking days during the 32-week double-blind period was significantly lower for psilocybin compared to placebo (mean difference of 13.9, 95% CI = 3.0–24.7, *p* = 0.01). In a pilot study (*n* = 15), the 7-day point prevalence of smoking abstinence at 26 weeks was 80% (12/15), and at 52 weeks 67% (10/15).

**Conclusion:**

Only one RCT and three small clinical trials were identified assessing the efficacy of psilocybin combined with some form of psychotherapy in patients with alcohol and tobacco use disorder. All four clinical trials indicated a beneficial effect of psilocybin-assisted therapy on SUD symptoms. Larger RCTs in patients with SUDs need to evaluate whether psilocybin-assisted therapy is effective in patients with SUD.

## Introduction

Globally, the 12-month prevalence of substance use disorder (SUD) is 2.2%, with alcohol use disorder as the most prevalent (1.5%) of all SUDs (excluding tobacco use disorder). The 12-month prevalence of SUDs is even higher among high-income countries (1). Alcohol use accounts for 4.2% of all disability-adjusted life-years (DALYs), while drug use accounts for 1.3% of all DALYs worldwide (2). In addition to the detrimental health consequences, there are also enormous economic consequences of SUD, with an estimated cost of $249 billion from alcohol, $300 billion from tobacco, and $193 billion from other drugs a year in the United States of America (USA) alone. Despite these adverse health and economic consequences of SUDs, therapeutic options are still limited. Food- and Drug Administration (FDA) approved medications are available for alcohol, tobacco, and opioid use disorders. Still, for other SUDs, such as cocaine and cannabis use disorders, no approved medications are available (3). Relapse rates remain high across all SUDs, with >75% of patients with primary cannabis and cocaine use disorder and >65% of primary alcohol and opioid use disorder showing relapse throughout 12 months after evidence-based treatment (4). Arguably, relapse rates are even higher in non-substance-related disorders, as >90% of patients with gambling disorder who had recently quit gambling showed relapse throughout 12 months (5). This means, there is an unmet need for effective treatments of SUD and non-substance-related disorders.

Psychedelics [e.g., with lysergic acid diethylamide (LSD)] have shown promising results as treatment of SUDs. In the majority of studies, some form of psychotherapy was given in combination with the psychedelic treatment, but not in all, which is usually referred to as psychedelic-assisted therapy (6). Psychedelic-assisted therapy comprises three stages: (1) preparation; (2) the psychedelic session; and (3) integration. Preparation is thought to be vital for maximizing the potential benefit from the psychedelic session, while integration is believed to be important for prolonging the improvements (7). Currently, interest in the efficacy of psychedelic treatment for SUDs is increasing (8). The term “psychedelic” was first proposed by Oswald in 1957 and comes from the Greek words “psyche” (i.e., mind) and “deloun” (i.e., manifesting) and refers to the subjective effects of these agents. A categorization of psychedelics can be made based on their chemical structure into three different classes: (1) tryptamines (e.g., psilocybin); (2) ergolines (e.g., LSD); and (3) phenethylamines (e.g., mescaline) (9). All these agents are closely related to the endogenous neurotransmitter serotonin [i.e., 5-hydroxytryptamine (5-HT)] and induce their effect *via* activation of the 5-HT_2A_-receptors (10). Sometimes, a more broad definition of psychedelics is used, and dissociates, and deliriants (e.g., ketamine and ibogaine) are included as well, which have a different mechanism of action (9). Swiss chemist dr. Albert Hoffman first synthesized LSD in 1938, (11) while he first identified psilocybin and its active metabolite psilocin as the psychoactive compounds of the psilocybe mushrooms in 1958. In the subsequent year, he synthesized psilocybin, which was later marketed as Indocybin by the pharmaceutical company Sandoz (12). Throughout the 1950s and 1960s, considerable research was conducted investigating the efficacy of LSD and psilocybin for various conditions, including anxiety, depression, and existential distress in patients with terminal cancer, in patients with opioid use disorder, and in patients with alcohol use disorder (6, 13). Possible therapeutic mechanisms of psychedelics might be at the biochemical, neural, and psychological level (14).

Krebs and Johansen (15) conducted a meta-analysis of randomized controlled trials (RCTs) evaluating the efficacy of LSD for alcohol use disorder. They identified *k* = 6 eligible trials, including *n* = 536 patients, and found a beneficial effect of LSD on alcohol use disorder with comparable efficacy to disulfiram (15). Savage and McCabe (16) also found a beneficial effect of LSD on heroin use disorder in *n* = 78 inmates of correctional institutions (16). However, in 1970, the Controlled Substances Act was signed into law by former USA President Richard Nixon placing psychedelics such as LSD and psilocybin in Schedule 1 (i.e., has a high potential for abuse, has no currently accepted medical use in treatment, and has a lack of safety for use under medical supervision), which hindered conducting psychedelic research (17). Other factors, such as negative publicity about psychedelics, doubts regarding their efficacy, and allocation of funding to related research fields (e.g., schizophrenia), increasingly discouraged research on psychedelics (18).

Recent years have seen a resurgence in psychedelic research, a development sometimes referred to as the psychedelic renaissance. Since 2016, three systematic reviews have been conducted evaluating the efficacy of psychedelics (i.e., psilocybin, but also other classic serotonergic psychedelics such as LSD were included in these systematic reviews) as a treatment for psychiatric disorders (i.e., SUD, but also other psychiatric disorders such as depression and anxiety were included in these systematic reviews) (19–21). Although these reviews provide an excellent overview of clinical trials published in the last 25 years, including multiple psychedelics and conditions, they may have missed clinical trials evaluating the efficacy of psychedelics that were conducted before the 1980s (19–21). As shown by Fuentes et al. (6), many trials have been conducted assessing the efficacy of LSD in SUD in these early decades, which may have been an important era for psilocybin research as well (6). In addition, many trials evaluating the efficacy of psilocybin in SUDs have been started since 2016, some of which may already have been published and not yet included in previous systematic reviews. Therefore, we performed a systematic review without restrictions on publication date. Our aim was to assess the efficacy of psilocybin in patients with SUD and non-substance-related disorders. In addition, a quality assessment was done of the included clinical trials.

## Methods

### Search strategy

We performed a systematic literature search up to September 2, 2022 (in each database from the earliest published manuscript, which dates back to 1781 in the case of PubMed), in the electronic databases: PubMed, Embase, Web of Science, Cochrane Library, Emcare, PsychINFO, and Academic Search Premier. The Preferred Reporting Items for Systematic reviews and Meta-Analysis (PRISMA) guidelines served as guiding principles for reporting in our systematic review (22). The search included a combination of terms related to “psilocybin” and “addiction.” The complete search strategy can be found in the Supplementary material. Two authors (PM and JF) independently screened the articles by title and abstract and full-text articles were obtained for all potentially relevant articles. In case of disagreement between the two authors, a third author (AB) was consulted to decide whether or not the full-text article should be obtained. Subsequently, the full-text articles were reviewed for inclusion by the same two authors. The reference lists of these full-text articles were searched, but no additional studies were found. In addition, a systematic search was done in the electronic databases: clinicaltrials.gov, and clinicaltrialsregister.eu, to identify ongoing trials evaluating the efficacy of psilocybin in SUDs and non-substance-related disorders.

### Eligibility criteria

The following inclusion criteria were established: intervention with ≥1 dose of psilocybin; clinical trial (open-label [pilot] studies, single-blind, or double-blind [placebo-controlled] trials); diagnosis of a SUD or non-substance-related disorder [i.e., diagnosed by the general practitioner or by a structured clinical interview based on Diagnostic and Statistical Manual of Mental Disorders (DSM) or International Classification of Diseases (ICD) criteria]; an outcome assessing severity of substance use or abstinence; adult patients (≥18 years); ≥10 patients, and language of the manuscript was English, Spanish, Portuguese, Dutch, Germany, or French. The following studies were excluded: animal studies; experimental studies in healthy volunteers; observational studies; review papers; qualitative studies; opinion pieces or comments; letters or editorials; conference abstracts or posters; and case reports.

### Data extraction

From the included studies, the following data was collected: names of authors; year of publication; study design; number and characteristics of patients; characteristics of the psilocybin and psychotherapeutic intervention; and non-substance-related disorder or SUD-related outcome(s).

### Quality assessment

The Risk Of Bias In Non-randomized Studies—of Interventions (ROBINS-I) tool was used to assess the risk of bias in the included non-randomized studies. The assessment of each included study in the review followed the following six steps: (1) to specify the research question through consideration of a target trial; (2) to specify the outcome and result being assessed; (3) for the specified result, to examine how the confounders and co-interventions were addressed; (4) to answer signaling questions for the seven bias domains; (5) formulate risk of bias judgments for each of the seven bias domains; and (6) formulate an overall judgment on risk of bias for the outcome and result being assessed (low risk of bias, moderate risk of bias, serious risk of bias, critical risk of bias, or no information on which to base a judgment about risk of bias) (23). The revised Cochrane risk-of-bias tool for randomized trials (RoB 2) was used to assess the risk of bias in the included randomized studies. Comparable as to the ROBINS-I tool, the ROB 2 tool followed the following six steps: (1) specify results being assessed; (2) specify the effect of interest; (3) list the sources of information used to inform assessment; (4) answer signaling questions for the five bias domains; (5) judge risk of bias for each domain; and (6) judge overall risk of bias for the result being assessed (low risk of bias, some concerns, high risk of bias) (24). Quality assessment was based on the primary efficacy outcome in the included studies.

## Results

**FIGURE 1:**
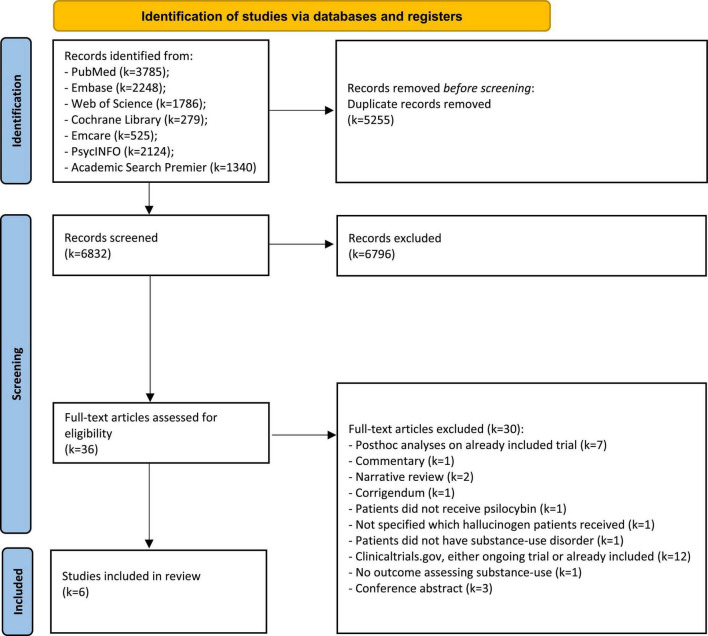
Flowchart of identified records and eventually included clinical trials.

We retrieved a total of *k* = 6832 unique records through our systematic search in various electronic databases (Figure 1). After screening titles and abstracts, *k* = 36 full-text articles were assessed for eligibility, and *k* = 6 articles were finally included in this systematic review. *K* = 2 articles were long-term follow-up results from the same clinical trial. All included clinical trials were conducted either in the USA or Poland.

A total of *k* = 130 registered clinical trials were identified through our systematic search in the two trial databases (Supplementary Figure 1). After screening, *k* = 11 ongoing trials on September 2, 2022, were included in this systematic review.

### Alcohol

Three studies were included assessing the efficacy of psilocybin-assisted therapy in patients with alcohol use disorder (Table 1). In the first study by Bogenschutz et al. (25), *n* = 10 patients were included with a diagnosis of active alcohol dependence according to the DSM, 4th edition (DSM-IV), ≥2 heavy drinking days [defined as a day of ≥5 standard drinks (i.e., 14 g of alcohol) for males and ≥4 standard drinks for females] in the past 30 days, who were concerned about their drinking, and not currently in treatment. Patients received two sessions with psilocybin at week 4 (21 mg/70 kg) and week 8 (28 mg/70 kg), and a psychosocial intervention comprising 12 sessions, 7 sessions of motivational enhancement therapy, 3 preparation sessions, and 2 debriefing sessions. The percentage of heavy drinking days decreased significantly between baseline and weeks 5–12 [mean difference of 26.0% (SD = 22.4), 95% CI = 8.7–43.2, *p* = 0.008]. Both percentage of drinking days and heavy drinking days remained significantly lower compared to baseline during the complete duration of follow-up of 36 weeks. Percentage of patients completely abstinent from alcohol was not reported. Treatment-related adverse effects were all mild, five patients reported headaches, while one patient reported nausea, diarrhea, and insomnia (25).

**TABLE 1:** Clinical trials evaluating the efficacy of psilocybin in substance-use disorders.

Article and country	Study design	Participants	Substance use disorder	Intervention	Control group	Follow-up	Primary outcome
Bogenschutz et al. (25), United States of America	Non-randomized	*N* = 10 (4 women and 6 men), mean age 40.1 years (SD = 10.3), mean duration alcohol use disorder 15.1 years (SD = 11.5), recruited using advertisements in local media	Alcohol	Psilocybin 21 mg/70 kg at week 4 and 28 mg/70 kg at week 8 + a psychosocial intervention (12 sessions)	No	36 weeks	Percentage heavy drinking days baseline vs. weeks 5–12
Bogenschutz et al. (26), United States of America	Randomized	*N* = 95 (42 women and 53 men), mean age 45.8 years (SD = 11.6), mean duration alcohol use disorder 14.2 years (SD = 9.7), recruited using advertisements in local media	Alcohol	Psilocybin 25 mg/70 kg- at week 4 and 25–40 mg/70 kg at week 8 + a psychosocial intervention (12 sessions)	Diphenhydramine 50 mg at week 4 and 50–100 mg at week 8 + a psychosocial intervention (12 sessions)	36 weeks	Percentage heavy drinking days over a 32-week period
Rydzyński et al. (27, 28), Poland	Non-randomized	*N* = 31 (31 men), mean age 37.9 years (SD unknown), mean duration of alcohol use disorder unknown, recruitment method unknown	Alcohol	Alternating psilocybin 6–30 mg (mean 15 sessions) and LSD 100–800 mcg (mean 12 sessions) in 5–7 day intervals + psychotherapy (number of sessions unknown)	No	Mean 6 years	Percentage abstinent from alcohol
Johnson et al. (29, 30), United States of America	Non-randomized	*N* = 15 (5 women and 10 men), mean age 51.0 years (SD = 10.5), mean duration of smoking 31.0 years (SD = 9.9), recruited using advertisements	Tobacco	Psilocybin 20 mg/70 kg at week 5; 30 mg/70 kg at week 7 and 9 + cognitive behavioral therapy (4 sessions)	No	52 weeks	Seven-day point prevalence abstinence at 26 and 52 weeks

SD, standard deviation.

In the second study by Bogenschutz et al. (26), a double-blind, placebo-controlled RCT, *n* = 95 patients with a DSM-IV diagnosis of active alcohol dependence, ≥4 heavy drinking days (same definition for heavy drinking day as in the first study was used) in the past 30 days and not currently in treatment were randomized 1:1 to either psilocybin or active placebo diphenhydramine. Patients in the psilocybin treatment arm received 2 sessions at week 4 (25 mg/70 kg) and week 8 (25–40 mg/70 kg), and a psychosocial intervention. Patients in the diphenhydramine treatment arm received two sessions at week 4 (50 mg) and week 8 (50–100 mg), and the same psychosocial intervention. The percentage of heavy drinking days during the 32-week double-blind period was significantly lower for psilocybin compared to diphenhydramine [mean 9.7 (SD = 26.2) vs. mean 23.6 (SD = 26.1), mean difference of 13.9, 95% CI = 3.0–24.7, Hedges *g* = 0.52, *p* = 0.01]. The percentage of patients completely abstinent from alcohol during the 32-week double-blind period did not differ significantly between psilocybin 22.9% vs. diphenhydramine 8.9% (OR = 3.1, 95% CI = 0.9–10.4, *p* = 0.06). Almost all patients and study therapists correctly guessed the treatment assignment at the first (94 and 92%) and the second (95 and 97%) session, respectively. No serious adverse events occurred in the psilocybin treatment arm (26).

In the third study by Rydzyński et al. (27, 28), *n* = 31 patients with alcohol use disorder were included with ≥1 failed quit attempt regarding their alcohol use despite treatment and not treated earlier with psilocybin and/or LSD. Patients first received psilocybin 6–30 mg (mean 12 mg), next LSD 100–800 mcg, and subsequently, for every 3 LSD sessions a psilocybin session. Each session had a 5–7 day interval. In addition, patients received psychotherapy. Eventually, treatment with LSD was abandoned and only psilocybin treatment was continued, because psilocybin had more favorable efficacy and fewer adverse effects, according to the authors. The percentage of patients that became completely abstinent from alcohol (mean duration of follow-up 6 years) was 32% (10/31), 32% (10/31) was abstinent from alcohol for 6–12 months, and 58% (18/31) of patients had a “satisfactory therapeutic effect,” which was not further defined by the authors. No treatment-related somatic nor psychiatric serious adverse effects were observed (27, 28).

At least 5 RCTs are ongoing assessing the efficacy of psilocybin in patients with alcohol use disorder, including a double-blind, placebo-controlled RCT, in patients with comorbid alcohol use disorder and major depressive disorder (Table 2).

**TABLE 2:** Clinical trials being conducted on September 2, 2022 evaluating the efficacy of psilocybin in substance-use disorders.

Principle investigator	Study design	Estimated enrolment	Substance use disorder	Intervention	Control group	Follow-up	Primary outcome(s)	ID
A. Fink-Jensen	RCT	*N* = 90	Alcohol	Single dose of psilocybin 25 mg + psychological support	Placebo (lactose) + psychological support	12 weeks	Change in percentage of heavy drinking days, from baseline until week 12	*^a^*NCT05416229
P. C. Nopoulos	RCT	*N* = 20	Alcohol	Single dose of psilocybin 30 mg + psychotherapy	Psychotherapy only	8 weeks	Change in daily alcohol use over the course of 8 weeks	*^a^*NCT05421065
K. Preller	RCT	*N* = 60	Alcohol	Single dose of psilocybin 25 mg	Placebo (mannitol)	26 weeks	Change in alcohol use behavior (i.e., daily alcohol use), from baseline until 6 months after intervention	*^a^*NCT04141501
F. S. Barrett	RCT	*N* = 90	Alcohol and major depressive disorder	Single dose of psilocybin 25 mg + psychotherapy	Placebo (microcrystalline cellulose) + psychotherapy	52 weeks	Change in percentage days abstinent/heavy drinking days, from baseline until 3 months after first drug intervention	*^a^*NCT04620759
PSY-CLA Study Contact	RCT	*N* = 160	Alcohol	Psilocybin 25 mg (unclear single or multiple doses) + psychotherapy	Placebo + psychotherapy	24 weeks	Mean number of heavy drinking days over 8 week treatment period	^b^2021-006200-33
M. W. Johnson	RCT	*N* = 95	Tobacco	Single dose of psilocybin 30 mg + psychotherapy	Nicotine replacement therapy + psychotherapy	52 weeks	Urinary cotinine/breath carbon monoxide, at 3, 6, and 12 months follow-up	*^a^*NCT01943994
M. W. Johnson	RCT	*N* = 66	Tobacco	Two doses of psilocybin 30 mg and 30–40 mg 1 week apart + psychotherapy	Two doses of niacin 150 mg and 150–200 mg 1 week apart + psychotherapy	52 weeks	Seven-day point prevalence abstinence at 52 weeks	*^a^*NCT05452772
M. W. Johnson	RCT	*N* = 92	Opioids	Single dose of psilocybin 40 mg + methadone maintenance program	Placebo + methadone maintenance program	12 weeks	Change in non-methadone opioid use, from baseline until 3 months after first drug intervention	*^a^*NCT05242029
R. Brown	Non-randomized	*N* = 10	Opioids	Two doses of psilocybin 4 weeks apart + (preparatory) counseling	No	9 weeks	Mean change in symptoms of opioid withdrawal up to 5 weeks	*^a^*NCT04161066
P. Hendricks	RCT	*N* = 40	Cocaine	Single dose of psilocybin 0.36 mg/kg + psychotherapy	Diphenhydramine 100 mg + psychotherapy	24 weeks	Difference in percentage days abstinent from cocaine/difference in complete sustained abstinence from cocaine/time to cocaine relapse	*^a^*NCT02037126
C. Stauffer	RCT	*N* = 30	Methamphetamine	Two doses of psilocybin (25 and 30 mg) 2 weeks apart + psychotherapy	Treatment-as-usual	32 weeks	Methamphetamine use (secondary outcome), from baseline until 2 months after discharge from admission	*^a^*NCT04982796

*^a^*ClinicalTrials.gov.

*^b^*Clinicaltrialsregister.eu.

### Tobacco

One study by Johnson et al. (29, 30) was included assessing the efficacy of psilocybin-assisted therapy in *n* = 15 patients who met the following criteria: smoking ≥10 cigarettes per day, being healthy as determined by medical interview, multiple unsuccessful past quit attempts, and the desire to quit smoking. The intervention consisted of three psilocybin sessions at week 5 (0.3 mg/kg), week 7, and week 9 (both 0.4 mg/kg), and four cognitive behavioral therapy sessions. Seven-day point prevalence of abstinence from smoking at 26-week follow-up was 80% (12/15) based on both biomarkers assessing smoking status and self-report outcome measures (29). At 52 weeks, 67% (10/15) of patients were confirmed abstinent from smoking, and at long-term follow-up [≥16 months, mean interval of 30 months (range = 16–57 months)] 60% (9/15) of patients was abstinent (30). All reported treatment-related adverse effects were mild. Six patients reported fear, fear of insanity, or feeling trapped during the psilocybin session, and eight patients reported headaches.

Two RCTs with a follow-up duration of 52 weeks are being conducted, assessing the efficacy of a single dose of psilocybin vs. nicotine replacement and two doses of psilocybin vs. placebo (niacin), respectively.

### Opioids

No studies were identified that evaluated the efficacy of psilocybin in patients with opioid use disorder. Currently, a double-blind, placebo-controlled RCT assessing the efficacy of psilocybin for opioid use disorder in patients on opioid agonist (methadone) treatment with ongoing opioid use and an open-label pilot study assessing the efficacy of psilocybin in patients with opioid use disorder on buprenorphine/naloxone treatment is being conducted.

### Cocaine

No studies were identified evaluating the efficacy of psilocybin in patients with cocaine use disorder. A pilot double-blind, placebo-controlled RCT assessing the efficacy of psilocybin for cocaine use disorder and MRI assessment to determine a potential biological mechanism of psilocybin’s effect is ongoing.

### Amphetamine and its derivatives

No studies were identified evaluating the efficacy of psilocybin in patients with amphetamine (or derivatives) use disorder. An RCT evaluating the efficacy of psilocybin-enhanced psychotherapy in patients with methamphetamine use disorder is currently recruiting participants with its primary aim of assessing acceptability, feasibility, and safety.

### Benzodiazepines or hypnotics, caffeine, cannabis, hallucinogens, ketamine, inhalants, or other (or unknown) substances, gambling, and gaming

No studies were identified evaluating the efficacy of psilocybin in patients with benzodiazepines or hypnotics, caffeine, cannabis, hallucinogens, ketamine, inhalants or other (or unknown) substances use disorder, nor in patients with a gambling or gaming disorder.

### Quality assessment of studies

The risk of bias has been assessed in the three non-randomized (Table 3) and the one randomized (Table 4) clinical trials (Supplementary Table 1). The three non-randomized trials were assessed as having a serious to critical risk of bias, and the randomized clinical trial was assessed as having some concerns of risk of bias.

**TABLE 3:** Quality assessment of the included non-randomized intervention studies based on the risk of bias.

References	Confounding	Selection of patients	Classification of interventions	Deviations from intended interventions	Missing data	Measurement of outcomes	Selection of reported results	Overall risk of bias
Bogenschutz et al. (25)	Serious	Low	Low	Low	Moderate	Serious	Low	Serious
Rydzyński et al. (27, 28)	Critical	Low	Low	Low	Low	Serious	Serious	Critical
Johnson et al. (29, 30)	Serious	Low	Low	Low	Low	Moderate	Low	Serious

Classifications of quality: low, moderate, serious, critical risk of bias, or no information.

**TABLE 4:** Quality assessment of the included randomized intervention studies based on the risk of bias.

Reference	Randomization process	Deviations from intended interventions	Missing outcome data	Measurement of the outcome	Selection of the reported results	Overall risk of bias
Bogenschutz et al. (26)	Low	Low	Low	Some concerns	Low	Some concerns

Classifications of quality: low, some concerns, high risk of bias.

## Discussion

In this systematic review, four clinical trials (*n* = 151 patients) assessing the efficacy of psilocybin in SUD were included, of which three clinical trials were in patients with alcohol use disorder and one clinical trial was in patients with tobacco use disorder. One of the trials in patients with alcohol use disorder was a double-blind, placebo-controlled RCT; the other three trials were single-arm pilot studies with a substantial risk of bias. All four clinical trials, which combined psilocybin with some form of psychotherapy, provided evidence for a significant beneficial effect of psilocybin-assisted therapy in patients with either alcohol or tobacco use disorder. These encouraging results have resulted in several (double-blind, placebo-controlled) RCTs currently ongoing in alcohol, tobacco, cocaine, opioid, and methamphetamine use disorders. Notable is the lack of trials evaluating the efficacy of psilocybin in patients with cannabis use disorder, despite the high prevalence and the lack of any approved medications for this condition.

Fuentes et al. (6) showed in their systematic review that eight eligible RCTs evaluating the efficacy of LSD in patients with alcohol use disorder and one eligible RCT in patients with opioid use disorder were conducted in the 1950s, 1960s, and 1970s (6). Krebs and Johansen (15) identified 33 clinical trials, of which only six met modern-day criteria of randomization, blinding, and outcome assessment (15). This is in stark contrast with the number of clinical trials evaluating the efficacy of psilocybin in patients with SUD before the 1980s, which was only one, and was contrary to our expectations. One trial evaluating psilocybin in patients with alcohol use disorder conducted in the 1960s was excluded from this systematic review. It did not include an outcome assessing severity of substance use or abstinence, but only evaluated treatment-related adverse effects (31). Three trials included in this systematic review were conducted in the 21st century in the era after the Controlled Substances Act was signed in 1970, while no trial has been conducted in the past four decades evaluating the efficacy of LSD in SUD (6). Although it has been claimed that >2000 publications have been published evaluating psychedelics in conditions such as SUD, it seems that the majority used LSD as intervention, not psilocybin (32, 33). Both LSD and psilocybin primarily act *via* stimulation of the 5-HT_2A_-receptors, but LSD exhibits additional affinity for dopaminergic and α-adrenergic receptors (34). The acute effects of LSD (100 and 200 μg) were directly compared to psilocybin (15 and 30 mg) in a double-blind, placebo-controlled RCT in healthy volunteers. The two psychedelics showed qualitatively, and quantitatively very similar subjective effects and differences in effect are dose-dependent rather than compound-dependent. The onset of effects is significantly earlier, and the duration is substantially longer in LSD compared to psilocybin (35). A dose of 30 mg psilocybin, which is comparable to the dosages used by Bogenschutz et al. (25), Bogenschutz et al. (26), and Johnson et al. (29), (25, 26, 29) was found similar to 100 and 200 μg LSD (35). However, in the meta-analysis by Krebs and Johansen (15), which showed a beneficial effect of LSD on alcohol use disorder, 83% (5/6) of included trials used a single fixed dose of ≥450 μg LSD with a maximum of 800 μg LSD (15). It is unclear whether higher and/or multiple dosages translate to a more favorable effect than lower and/or single dosages of psilocybin or LSD. In addition, all the included studies in this systematic review adjusted psilocybin dose to body weight, (25–30) although there is no clear benefit for this adjustment and fixed dosing is more practical in clinical trials (35, 36).

Proposed working mechanisms of psychedelics, including psilocybin, for improving psychiatric symptoms such as depression and SUD are both biological (e.g., by inducing brain neuroplasticity through elevating Brain-Derived Neurotrophic Factor (BDNF) levels, which are diminished in psychiatric conditions) (34) and psychological (e.g., the degree to which a patient has a mystical-type experience during the psilocybin session seems an important mediator for the enduring effect of the psilocybin) (25, 37). The latter proposed working mechanism seems especially important in SUD as the mystical-type experience seems to induce behavioral change in a patient with SUD. This working mechanism would also explain why psychedelics seem effective in different SUDs as opposed to the currently approved pharmacological treatments for SUD, which have a different working mechanism and are, in most cases, only effective in a specific SUD (e.g., disulfiram in alcohol use disorder).

In the design of clinical trials (with psilocybin treatment), several important issues need to be taken into account, including an adequate control group, randomization, blinding, and generalizability of the results. Regulatory agencies even recognize single-arm trials with external historical controls to assess promising treatments for specific indications. However, three trials in this systematic review were non-randomized, had no adequate control group, and were therefore prone to bias. Preferably, future trials are double-blind, placebo-controlled RCTs to account for a potential placebo effect. Most tobacco use disorder patients had a past use of psychedelics, which could have influenced the expectancy of these patients regarding the efficacy of psilocybin and have thus an effect on the results (29). Pretreatment-positive expectations about psychedelic microdosing predicted improvements in psychiatric symptoms (38). A meta-analysis by Wilkinson et al. (39) evaluating the efficacy of ketamine for major depressive disorder or bipolar disorder showed a dramatic reduction in effect size when comparing the impact of an inactive (saline) to the active placebo midazolam (effect size *d* = 1.8, 95% CI = 1.4–2.2 vs. *d* = 0.7, 95% CI = 0.4–0.9) (39). Despite comparing psilocybin to an active placebo, Bogenschutz et al. (26) still observed a medium effect size for the efficacy of psilocybin. However, even in a double-blind, placebo-controlled RCT, this problem with expectancy effects, a key contributor to the placebo response, is not easily accounted for given >90% of patients and study therapists correctly guessed treatment assignment to either psilocybin or the active placebo diphenhydramine (26). Maintaining successful masking remains a major challenge in psychedelic clinical trials for which there is not yet an adequate solution. Another challenging problem is the generalizability of the results. Johnson et al. (29) screened *n* = 323 individuals, and only *n* = 15 (5%) patients were eventually included. (29) Bogenschutz et al. (25) screened *n* = 70 individuals, and only *n* = 10 (14%) patients were eventually included (25). Bogenschutz et al. (26) screened *n* = 569 individuals, and only *n* = 95 (17%) were eventually randomized (26). From a safety point of view strict in- and exclusion criteria makes sense. Still, a risk is that external validity is relatively low and that diminished effects will be observed with psilocybin in a therapeutic setting (33). Coadministration of an extensive psychological treatment (motivational enhancement therapy and cognitive behavioral therapy) during the clinical trials makes interpretation of the treatment effect of psilocybin on alcohol and tobacco use disorder even more difficult (25, 26, 29). Ideally, an RCT would compare four different treatment arms as in the RCT by Grabski et al. (40) evaluating the efficacy of adjunctive ketamine in patients with alcohol use disorder to separate the different treatment effects. In this double-blind, placebo-controlled RCT, the four treatment arms were: (1) ketamine + psychotherapy; (2) placebo + psychotherapy; (3) ketamine + alcohol education (therapy control); and (4) placebo + alcohol education. The greatest reduction in alcohol use was seen in the ketamine + psychotherapy group vs. placebo + alcohol education (mean difference of 15.9, 95% CI = 3.8–28.1) (40). It seems the eventual treatment strategy will likely consist of psilocybin combined with psychotherapy, given the latter seems to be needed to derive meaning from the psilocybin sessions and incorporate insights from the psilocybin sessions into daily life and change current substance use behavior. However, alternative hypotheses assume more direct pharmacological working mechanism of psychedelics, and thus microdosing would be a potential treatment eliminating the need for a psychedelic experience. However, so far, results with psychedelic microdosing are less promising (41).

Currently, > 10 clinical trials are registered evaluating the efficacy of psilocybin in SUD, most are in alcohol use disorder. Other patient populations include tobacco, cocaine, opioids, and methamphetamine use disorder. Conducting clinical trials in alcohol and tobacco use disorder can be understood from their high prevalence worldwide. It is less clear why no clinical trials in cannabis use disorder are currently conducted as it is the most common drug use disorder worldwide, especially among youth (42). Other interesting patient populations to study the efficacy of psilocybin would be benzodiazepines or hypnotics use disorder and gambling disorder, for which there is a pressing need for effective treatments. Almost all registered trials have a control group and are randomized. The risk of confounding, which was serious to critical in the included non-randomized studies in this systemic review, is minimized. The registered RCT by Johnson (ClinicalTrials.gov Identifier: NCT01943994) is of special interest, evaluating the efficacy of psilocybin combined with psychotherapy versus an approved pharmacological treatment (i.e., nicotine replacement therapy) combined with psychotherapy. Here, psilocybin will be compared for the first time with an approved active pharmacological treatment (in patients with SUD). Maintaining masking in double-blind, placebo-controlled RCTs evaluating psychedelics is usually unsuccessful, which may result in an increased effect size for the efficacy of psilocybin. It is essential to know how psilocybin compares to approved pharmacological treatments and its effect size in comparative efficacy trials. Given that psychological treatments have high relapse rates in the long term, the limited number of available approved pharmacological interventions for SUD, and the high adverse health and economic consequences of SUDs, alternative treatment options are highly warranted for this population. Psilocybin has shown promising first results in SUD treatment that should be replicated and established in larger trials. Questions whether psychotherapy is a necessary treatment component for psilocybin to be effective and if so, what form of psychotherapy is indicated, and whether higher and/or multiple dosages translate to a more favorable effect compared to lower and/or single dosages of psilocybin are not addressed in the currently registered clinical trials. The results of ongoing and future studies assessing the efficacy of psychedelics in SUD will reveal their true potential.

## Conclusion

In this systematic review, we identified only one double-blind, placebo-controlled RCT, and three small clinical trials, of which three were conducted in the 21st century assessing the efficacy of psilocybin in patients with alcohol and tobacco use disorder. All four studies combined psilocybin with some form of psychotherapy and showed a beneficial effect of psilocybin-assisted therapy on SUD, but the risk of bias ranged from some concerns to critical. Future (double-blind, placebo-controlled) RCTs in patients with SUD need to evaluate whether psilocybin-assisted therapy is effective in this population.

## Data availability statement

The original contributions presented in this study are included in the article/Supplementary material, further inquiries can be directed to the corresponding author.

## Author contributions

PM and AB designed the study. PM and JF reviewed the abstracts and the manuscripts, obtained the data from the selected manuscripts, and performed the quality assessment of the included manuscripts. PM wrote the first and successive versions of the manuscript. PM, JF, AK, JS, MW, AG, KK, AS, MS, MB, and AB contributed to the interpretation of the results, critical revisions to the drafts of the manuscript, and approved the final version. All authors contributed to the article and approved the submitted version.
