# Supplementary material for: Therapeutic effect of psilocybin in addiction: A systematic review
Source: Front Psychiatry. 2023 Feb 9;14:1134454. doi: 10.3389/fpsyt.2023.1134454 (PMC9947277; doi:10.3389/fpsyt.2023.1134454)
Supplement: Supplementary file 1 [file Data_sheet_1.docx]

**Supplementary**

**Therapeutic effect of psilocybin in addiction: a systematic review**

Pim B. van der Meer, Juan J. Fuentes, Ad A. Kaptein, Jan W. Schoones, Marleen M. de Waal, Anneke E. Goudriaan, Kees Kramers, Arnt Schellekens, Metten Somers, Matthijs G. Bossong, Albert Batalla

**Contents**

**Supplementary Table 1.** Quality assessment of studies in detail

**Supplementary Figure 1.** Flowchart of identified trials and eventually included trials

**Complete search strategy**

**Table 1.** Quality assessment of studies in detail

| **Domains** | **Risk of bias** | **Reason** |
| --- | --- | --- |
| **Bogenschutz et al. (2015)** | | |
| Confounding | Serious | We are aware that the risk of bias for the domain confounding cannot be assessed optimally in a single-arm clinical trial. Certain important potential confounders were assessed (e.g., self-efficacy). However, patients were recruited through advertisements making it likely patients participating had a (high) positive expectancy with regard to psilocybin treatment and therefore affecting the outcomes. |
| Selection of patients | Low | All patients who would have been eligible for the target trial were included in the study and start of intervention and follow-up coincided. |
| Classification of interventions | Low | The intervention group was clearly defined and classification of intervention status was solely based on information collected at the time of intervention. |
| Deviations from intended interventions | Low | Deviations from intended intervention reflected usual practice and were unlikely to impact the outcome. |
| Missing data | Moderate | Data was reasonably complete, but no sensitivity analyses was done regarding the excluded patient as relapsed. |
| Measurement of outcomes | Serious | Patients and people delivering the interventions were aware of the patients’ assigned intervention during the trial. The primary outcome was assessed only with the subjective time-line follow-back method. |
| Selection of reported results | Low | There is clear evidence that reported results correspond to the intended outcomes and analyses in the protocol at clinicaltrials.gov. |
| Overall risk of bias | Serious | Serious risk of bias with confounding and measurement of outcomes, meaning overall risk of bias is classified as serious risk of bias. |
| **Rydzyński et al. (1968) and (1978)** | | |
| Confounding | Critical | We are aware that the risk of bias for the domain confounding cannot be assessed optimally in a single-arm clinical trial. No potential confounders were assessed and it was unclear how patients were recruited. |
| Selection of patients | Low | Selection of patients into the study was not based on patient characteristics observed after start of treatment and start of intervention and follow-up coincided. |
| Classification of interventions | Low | The intervention group was clearly defined and classification of intervention status was solely based on information collected at the time of intervention. |
| Deviations from intended interventions | Low | Deviations from intended intervention reflected usual practice and were unlikely to impact the outcome. |
| Missing data | Low | Data was reasonably complete and missing data was addressed appropriately. |
| Measurement of outcomes | Serious | Patients and people delivering the interventions were aware of the patients’ assigned intervention during the trial. It is unclear how the outcomes were assessed. |
| Selection of reported results | Serious | There is a high risk of selective reporting from among multiple analyses, as no pre-specified analysis plan was reported and no primary outcome described. |
| Overall risk of bias | Critical | Critical risk of bias with confounding, meaning overall risk of bias is classified as critical risk of bias. |
| **Johnson et al. (2014) and (2017)** | | |
| Confounding | Serious | We are aware that the risk of bias for the domain confounding cannot be assessed optimally in a single-arm clinical trial. Certain important potential confounders were assessed (e.g., self-efficacy). However, patients were recruited through advertisements making it likely patients participating had a (high) positive expectancy with regard to psilocybin treatment and therefore affecting the outcomes. |
| Selection of patients | Low | All patients who would have been eligible for the target trial were included in the study and start of intervention and follow-up coincided. |
| Classification of interventions | Low | The intervention group was clearly defined and classification of intervention status was solely based on information collected at the time of intervention. |
| Deviations from intended interventions | Low | Deviations from intended intervention reflected usual practice and were unlikely to impact the outcome. |
| Missing data | Low | Data was reasonably complete and missing data was addressed appropriately. |
| Measurement of outcomes | Moderate | Patients and people delivering the interventions were aware of the patients’ assigned intervention during the trial. The primary outcome was assessed with the subjective time-line follow-back method and the objective exhaled carbon monoxide and urinary cotinine level method. |
| Selection of reported results | Low | There is clear evidence that reported results correspond to the intended outcomes and analyses in the protocol at clinicaltrials.gov. |
| Overall risk of bias | Serious | Serious risk of bias with confounding, meaning overall risk of bias is classified as serious risk of bias. |
| **Bogenschutz et al. (2022)** | | |
| Randomization process | Low | Allocation sequence was random, concealed, and no differences between the two groups suggested a problem with randomization process. |
| Deviations from intended interventions | Low | Patients and people delivering the interventions were aware of the patients’ assigned intervention during the trial, but there were no deviations from the intended intervention. |
| Missing outcome data | Low | Outcome data were obtained for almost all patients except for ethylglucuronide concentration. However, reasons for missing ethylglucuronide data was due to valid reasons and could not depend on its true value. |
| Measurement of the outcome | Some concerns | Almost all patients and people delivering the interventions were aware of the patients’ assigned intervention during the trial, but it is unlikely that this influenced the primary outcome. |
| Selection of the reported results | Low | Reported primary outcome was in accordance with the pre-specified analysis plan in the protocol, was unlikely to have been selected from multiple eligible outcome measurements within the outcome domain and unlikely to have been selected on the basis of the results. |
| Overall risk of bias | Some concerns | Some concerns with measurement of outcome, while all other domains had a low risk, meaning overall risk of bias is classified as some concerns. |

**Supplementary Figure 1.** Flowchart of identified trials and eventually included trials

**Identification of studies via databases and registers**

Trials identified from:

- Clinicaltrials.gov (k=113)

- Clinicaltrialsregister.eu (k=17)

**Identification**

Records screened

(k=130)

**Screening**

Trials excluded (k=119):

- Evaluating efficacy of psilocybin in SUD, but registered in both databases (k=1)

- Not evaluating efficacy of psilocybin in SUD, but only safety or feasibility (k=2)

- Not evaluating psilocybin in SUD, but in other disorders or diseases (k=116)

Trials assessed for eligibility

(n=130)

Trials included in review

(k=11)

**Included**

**Search strategy**

Pubmed

(("Psilocybin"[Mesh] OR "Psilocybin"[tw] OR "Psilocybins"[tw] OR "Psilocybin*"[tw] OR "Psilocibin"[tw] OR "Psilocibin*"[tw] OR "Psilocybe"[tw] OR "Psilocybe"[tw] OR "psilocin"[Supplementary Concept] OR "Psilocin"[tw] OR "Psilocin*"[tw] OR "omega-N-methyl-4-hydroxytryptamine"[Supplementary Concept] OR "omega-N-methyl-4-hydroxytryptamine"[tw] OR "norpsilocin"[tw] OR "norpsilocin*"[tw] OR "Hallucinogens"[Mesh] OR "hallucinogens"[tw] OR "hallucinogen*"[tw] OR "psychedelic"[tw] OR "psychedel*"[tw]) AND ("antiaddictive"[tw] OR "antiaddict*"[tw] OR "addict"[tw] OR "addict*"[tw] OR "addicted"[tw] OR "addiction medicine"[mesh] OR "addiction"[tw] OR "addictions"[tw] OR "addicts"[tw] OR "alcohol abstinence"[mesh] OR "alcohol drinking"[mesh] OR "alcohol withdrawal"[tw] OR "alcohol"[tw] OR "alcohol*"[tw] OR "alcoholic intoxication"[tw] OR "alcohol-induced disorders"[mesh] OR "alcohol-induced disorders"[tw] OR "alcoholism"[tw] OR "alcohol-related disorders"[mesh] OR "alcohols"[mesh] OR "amphetamine"[mesh] OR "amphetamine"[tw] OR "amphetamine*"[tw] OR "amphetamine-related disorders"[mesh] OR "amphetamines"[mesh] OR "analgesics, opioid"[mesh] OR "behavior, addictive"[mesh] OR "benzodiazepine"[tw] OR "benzodiazepine*"[tw] OR "benzodiazepines"[mesh] OR "benzodiazepines"[tw] OR "binge drinking"[tw] OR "cannabin*"[tw] OR "cannabis"[mesh] OR "cannabis"[tw] OR "cigar"[tw] OR "cigar*"[tw] OR "cigarette"[tw] OR "cigarette*"[tw] OR "cocaine smoking"[mesh] OR "cocaine"[mesh] OR "cocaine"[tw] OR "cocaine*"[tw] OR "cocaine-related disorders"[mesh] OR "crack cocaine"[mesh] OR "crack*"[tw] OR "dextroamphetamine"[mesh] OR "drug overdose"[tw] OR "ethanol"[mesh] OR "ethanol*"[tw] OR "gambl*"[tw] OR "gambling"[mesh] OR "heroin dependence"[mesh] OR "heroin"[tw] OR "heroin*"[tw] OR "inhalant abuse"[tw] OR "ketamine"[mesh] OR "ketamine*"[tw] OR "marijuana smoking"[mesh] OR "marijuana smoking"[tw] OR "marijuana"[tw] OR "marijuana*"[tw] OR "methadon*"[tw] OR "methamphetamine"[mesh] OR "morphine dependence"[mesh] OR "morphine derivatives"[mesh] OR "morphine"[mesh] OR "morphine"[tw] OR "morphine*"[tw] OR "narcotic"[tw] OR "narcotic*"[tw] OR "narcotic-related disorders"[mesh] OR "narcotics"[mesh] OR "National Institute on Alcohol Abuse and Alcoholism (U.S.)"[mesh] OR "neonatal abstinence syndrome"[tw] OR "nicotin*"[tw] OR "nicotine"[mesh] OR "opiate"[tw] OR "opiate*"[tw] OR "opioid"[tw] OR "opioid*"[tw] OR "opioid-related disorders"[mesh] OR "opioids"[tw] OR "opium dependence"[tw] OR "opium"[tw] OR "opium*"[tw] OR "phencyclidine"[tw] OR "phencyclidine*"[tw] OR "smoking"[tw] OR "substance abus*"[tw] OR "substance abuse treatment centers"[mesh] OR "substance abuse"[tw] OR "substance abuse, intravenous"[mesh] OR "substance abuse, oral"[mesh] OR "substance withdrawal"[tw] OR "substance-induced"[tw] OR "substance-related disorders"[mesh] OR "tobacco products"[mesh] OR "tobacco smoking"[mesh] OR "tobacco use cessation"[mesh] OR "tobacco use disorder"[mesh] OR "tobacco"[mesh] OR "tobacco"[tw] OR "tobacco*"[tw] OR "dependence"[tw]) NOT (("Animals"[mesh] OR "veterinary"[ti] OR "rabbit"[ti] OR "rabbits"[ti] OR "animal"[ti] OR "animals"[ti] OR "mouse"[ti] OR "mice"[ti] OR "rodent"[ti] OR "rodents"[ti] OR "rat"[ti] OR "rats"[ti] OR "pig"[ti] OR "pigs"[ti] OR "porcine"[ti] OR "horse"[ti] OR "horses"[ti] OR "equine"[ti] OR "cow"[ti] OR "cows"[ti] OR "bovine"[ti] OR "goat"[ti] OR "goats"[ti] OR "sheep"[ti] OR "ovine"[ti] OR "canine"[ti] OR "dog"[ti] OR "dogs"[ti] OR "feline"[ti] OR "cat"[ti] OR "cats"[ti]) NOT ("Humans"[mesh] OR "Humans"[ti] OR "Human"[ti] OR "patient"[ti] OR "patients"[ti])) NOT (("Review"[ptyp] OR "review"[ti]) NOT ("systematic"[sb] OR "Clinical Study"[ptyp] OR "trial"[ti] OR "RCT"[ti])) NOT (("Infant"[mesh] OR "Infant"[ti] OR "Infants"[ti] OR "Child"[mesh] OR "child"[ti] OR "children"[ti] OR "Adolescent"[mesh] OR "adolescent"[ti] OR "adolescents"[ti] OR "adolescence"[ti]) NOT ("Adult"[mesh] OR "adult"[ti] OR "adults"[ti] OR "elderly"[ti])))

Embase

((exp *"Psilocybine"/ OR "Psilocybin".ti,ab OR "Psilocybins".ti,ab OR "Psilocybin*".ti,ab OR "Psilocibin".ti,ab OR "Psilocibin*".ti,ab OR "Psilocybe".ti,ab OR "Psilocybe".ti,ab OR exp *"psilocin"/ OR "Psilocin".ti,ab OR "Psilocin*".ti,ab OR "omega-N-methyl-4-hydroxytryptamine".ti,ab OR "norpsilocin".ti,ab OR "norpsilocin*".ti,ab OR *"psychedelic agent"/ OR "hallucinogens".ti,ab OR "hallucinogen*".ti,ab OR "psychedelic".ti,ab OR "psychedel*".ti,ab) AND ("antiaddictive".ti,ab OR "antiaddict*".ti,ab OR "addict".ti,ab OR "addict*".ti,ab OR "addicted".ti,ab OR exp *"addiction medicine"/ OR "addiction".ti,ab OR "addictions".ti,ab OR "addicts".ti,ab OR exp *"alcohol abstinence"/ OR exp *"drinking behavior"/ OR "alcohol withdrawal".ti,ab OR "alcohol".ti,ab OR "alcohol*".ti,ab OR "alcoholic intoxication".ti,ab OR exp *"alcoholism"/ OR "alcohol-induced disorders".ti,ab OR "alcoholism".ti,ab OR exp *"alcohol derivative"/ OR exp *"amphetamine"/ OR "amphetamine".ti,ab OR "amphetamine*".ti,ab OR exp "amphetamine dependence"/ OR exp *"narcotic analgesic agent"/ OR exp *"addiction"/ OR "benzodiazepine".ti,ab OR "benzodiazepine*".ti,ab OR exp *"benzodiazepine"/ OR "benzodiazepines".ti,ab OR "binge drinking".ti,ab OR "cannabin*".ti,ab OR exp *"cannabis"/ OR "cannabis".ti,ab OR "cigar".ti,ab OR "cigar*".ti,ab OR "cigarette".ti,ab OR "cigarette*".ti,ab OR exp *"cocaine smoking"/ OR "cocaine"/ OR "cocaine".ti,ab OR "cocaine*".ti,ab OR exp *"cocaine dependence"/ OR "crack*".ti,ab OR exp *"dexamphetamine"/ OR exp *"drug overdose"/ OR "drug overdose".ti,ab OR exp *"alcohol"/ OR "ethanol*".ti,ab OR "gambl*".ti,ab OR exp *"gambling"/ OR exp *"heroin dependence"/ OR "heroin".ti,ab OR "heroin*".ti,ab OR exp *"diamorphine"/ OR exp *"inhalant abuse"/ OR "inhalant abuse".ti,ab OR exp *"ketamine"/ OR "ketamine*".ti,ab OR exp *"cannabis smoking"/ OR "marijuana smoking".ti,ab OR "marijuana".ti,ab OR "marijuana*".ti,ab OR "methadon*".ti,ab OR exp *"methamphetamine"/ OR exp *"morphine addiction"/ OR exp *"morphine derivative"/ OR exp *"morphine"/ OR "morphine".ti,ab OR "morphine*".ti,ab OR "narcotic".ti,ab OR "narcotic*".ti,ab OR exp *"narcotic agent"/ OR exp *"neonatal abstinence syndrome"/ OR "neonatal abstinence syndrome".ti,ab OR "nicotin*".ti,ab OR exp *"nicotine"/ OR "opiate".ti,ab OR "opiate*".ti,ab OR "opioid".ti,ab OR "opioid*".ti,ab OR exp *"opiate addiction"/ OR "opioids".ti,ab OR "opium dependence".ti,ab OR "opium".ti,ab OR "opium*".ti,ab OR "phencyclidine".ti,ab OR "phencyclidine*".ti,ab OR exp *"smoking"/ OR "smoking".ti,ab OR "substance abus*".ti,ab OR exp *"drug dependence treatment"/ OR "substance abuse".ti,ab OR exp *"substance abuse"/ OR "substance withdrawal".ti,ab OR "substance-induced".ti,ab OR eexp *"drug dependence"/ OR exp *"tobacco"/ OR exp *"smoking cessation"/ OR exp *"tobacco dependence"/ OR "tobacco".ti,ab OR "tobacco*".ti,ab OR "dependence".ti,ab) NOT ((exp "Animals"/ OR "veterinary".ti OR "rabbit".ti OR "rabbits".ti OR "animal".ti OR "animals".ti OR "mouse".ti OR "mice".ti OR "rodent".ti OR "rodents".ti OR "rat".ti OR "rats".ti OR "pig".ti OR "pigs".ti OR "porcine".ti OR "horse".ti OR "horses".ti OR "equine".ti OR "cow".ti OR "cows".ti OR "bovine".ti OR "goat".ti OR "goats".ti OR "sheep".ti OR "ovine".ti OR "canine".ti OR "dog".ti OR "dogs".ti OR "feline".ti OR "cat".ti OR "cats".ti) NOT (exp "Humans"/ OR "Humans".ti OR "Human".ti OR "patient".ti OR "patients".ti)) NOT (("Review"/ OR "review".ti) NOT ("systematic review"/ OR exp "Clinical Study"/ OR "trial".ti OR "RCT".ti)) NOT ((exp "Infant"/ OR "Infant".ti OR "Infants".ti OR exp "Child"/ OR "child".ti OR "children".ti OR exp "Adolescent"/ OR "adolescent".ti OR "adolescents".ti OR "adolescence".ti) NOT (eexp "Adult"/ OR "adult".ti OR "adults".ti OR "elderly".ti)))

- - NOT conference review.pt
  - NOT (conference review or conference abstract).pt
  - AND (conference abstract).pt

Web of Science

((TI=("Psilocybine" OR "Psilocybin" OR "Psilocybins" OR "Psilocybin*" OR "Psilocibin" OR "Psilocibin*" OR "Psilocybe" OR "Psilocybe" OR "psilocin" OR "Psilocin" OR "Psilocin*" OR "omega N methyl 4 hydroxytryptamine" OR "norpsilocin" OR "norpsilocin*" OR "psychedelic agent" OR "hallucinogens" OR "hallucinogen*" OR "psychedelic" OR "psychedel*") OR AK=("Psilocybine" OR "Psilocybin" OR "Psilocybins" OR "Psilocybin*" OR "Psilocibin" OR "Psilocibin*" OR "Psilocybe" OR "Psilocybe" OR "psilocin" OR "Psilocin" OR "Psilocin*" OR "omega N methyl 4 hydroxytryptamine" OR "norpsilocin" OR "norpsilocin*" OR "psychedelic agent" OR "hallucinogens" OR "hallucinogen*" OR "psychedelic" OR "psychedel*") OR AB=("Psilocybine" OR "Psilocybin" OR "Psilocybins" OR "Psilocybin*" OR "Psilocibin" OR "Psilocibin*" OR "Psilocybe" OR "Psilocybe" OR "psilocin" OR "Psilocin" OR "Psilocin*" OR "omega N methyl 4 hydroxytryptamine" OR "norpsilocin" OR "norpsilocin*" OR "psychedelic agent" OR "hallucinogens" OR "hallucinogen*" OR "psychedelic" OR "psychedel*")) AND (TI=("antiaddictive" OR "antiaddict*" OR "addict" OR "addict*" OR "addicted" OR "addiction medicine" OR "addiction" OR "addictions" OR "addicts" OR "alcohol abstinence" OR "drinking behavior" OR "alcohol withdrawal" OR "alcohol" OR "alcohol*" OR "alcoholic intoxication" OR "alcoholism" OR "alcohol induced disorders" OR "alcoholism" OR "alcohol derivative" OR "amphetamine" OR "amphetamine" OR "amphetamine*" OR "amphetamine dependence" OR "narcotic analgesic agent" OR "addiction" OR "benzodiazepine" OR "benzodiazepine*" OR "benzodiazepine" OR "benzodiazepines" OR "binge drinking" OR "cannabin*" OR "cannabis" OR "cannabis" OR "cigar" OR "cigar*" OR "cigarette" OR "cigarette*" OR "cocaine smoking" OR "cocaine" OR "cocaine" OR "cocaine*" OR "cocaine dependence" OR "crack*" OR "dexamphetamine" OR "drug overdose" OR "drug overdose" OR "alcohol" OR "ethanol*" OR "gambl*" OR "gambling" OR "heroin dependence" OR "heroin" OR "heroin*" OR "diamorphine" OR "inhalant abuse" OR "inhalant abuse" OR "ketamine" OR "ketamine*" OR "cannabis smoking" OR "marijuana smoking" OR "marijuana" OR "marijuana*" OR "methadon*" OR "methamphetamine" OR "morphine addiction" OR "morphine derivative" OR "morphine" OR "morphine" OR "morphine*" OR "narcotic" OR "narcotic*" OR "narcotic agent" OR "neonatal abstinence syndrome" OR "neonatal abstinence syndrome" OR "nicotin*" OR "nicotine" OR "opiate" OR "opiate*" OR "opioid" OR "opioid*" OR "opiate addiction" OR "opioids" OR "opium dependence" OR "opium" OR "opium*" OR "phencyclidine" OR "phencyclidine*" OR "smoking" OR "smoking" OR "substance abus*" OR "drug dependence treatment" OR "substance abuse" OR "substance abuse" OR "substance withdrawal" OR "substance induced" OR e"drug dependence" OR "tobacco" OR "smoking cessation" OR "tobacco dependence" OR "tobacco" OR "tobacco*" OR "dependence") OR AK=("antiaddictive" OR "antiaddict*" OR "addict" OR "addict*" OR "addicted" OR "addiction medicine" OR "addiction" OR "addictions" OR "addicts" OR "alcohol abstinence" OR "drinking behavior" OR "alcohol withdrawal" OR "alcohol" OR "alcohol*" OR "alcoholic intoxication" OR "alcoholism" OR "alcohol induced disorders" OR "alcoholism" OR "alcohol derivative" OR "amphetamine" OR "amphetamine" OR "amphetamine*" OR "amphetamine dependence" OR "narcotic analgesic agent" OR "addiction" OR "benzodiazepine" OR "benzodiazepine*" OR "benzodiazepine" OR "benzodiazepines" OR "binge drinking" OR "cannabin*" OR "cannabis" OR "cannabis" OR "cigar" OR "cigar*" OR "cigarette" OR "cigarette*" OR "cocaine smoking" OR "cocaine" OR "cocaine" OR "cocaine*" OR "cocaine dependence" OR "crack*" OR "dexamphetamine" OR "drug overdose" OR "drug overdose" OR "alcohol" OR "ethanol*" OR "gambl*" OR "gambling" OR "heroin dependence" OR "heroin" OR "heroin*" OR "diamorphine" OR "inhalant abuse" OR "inhalant abuse" OR "ketamine" OR "ketamine*" OR "cannabis smoking" OR "marijuana smoking" OR "marijuana" OR "marijuana*" OR "methadon*" OR "methamphetamine" OR "morphine addiction" OR "morphine derivative" OR "morphine" OR "morphine" OR "morphine*" OR "narcotic" OR "narcotic*" OR "narcotic agent" OR "neonatal abstinence syndrome" OR "neonatal abstinence syndrome" OR "nicotin*" OR "nicotine" OR "opiate" OR "opiate*" OR "opioid" OR "opioid*" OR "opiate addiction" OR "opioids" OR "opium dependence" OR "opium" OR "opium*" OR "phencyclidine" OR "phencyclidine*" OR "smoking" OR "smoking" OR "substance abus*" OR "drug dependence treatment" OR "substance abuse" OR "substance abuse" OR "substance withdrawal" OR "substance induced" OR e"drug dependence" OR "tobacco" OR "smoking cessation" OR "tobacco dependence" OR "tobacco" OR "tobacco*" OR "dependence") OR AB=("antiaddictive" OR "antiaddict*" OR "addict" OR "addict*" OR "addicted" OR "addiction medicine" OR "addiction" OR "addictions" OR "addicts" OR "alcohol abstinence" OR "drinking behavior" OR "alcohol withdrawal" OR "alcohol" OR "alcohol*" OR "alcoholic intoxication" OR "alcoholism" OR "alcohol induced disorders" OR "alcoholism" OR "alcohol derivative" OR "amphetamine" OR "amphetamine" OR "amphetamine*" OR "amphetamine dependence" OR "narcotic analgesic agent" OR "addiction" OR "benzodiazepine" OR "benzodiazepine*" OR "benzodiazepine" OR "benzodiazepines" OR "binge drinking" OR "cannabin*" OR "cannabis" OR "cannabis" OR "cigar" OR "cigar*" OR "cigarette" OR "cigarette*" OR "cocaine smoking" OR "cocaine" OR "cocaine" OR "cocaine*" OR "cocaine dependence" OR "crack*" OR "dexamphetamine" OR "drug overdose" OR "drug overdose" OR "alcohol" OR "ethanol*" OR "gambl*" OR "gambling" OR "heroin dependence" OR "heroin" OR "heroin*" OR "diamorphine" OR "inhalant abuse" OR "inhalant abuse" OR "ketamine" OR "ketamine*" OR "cannabis smoking" OR "marijuana smoking" OR "marijuana" OR "marijuana*" OR "methadon*" OR "methamphetamine" OR "morphine addiction" OR "morphine derivative" OR "morphine" OR "morphine" OR "morphine*" OR "narcotic" OR "narcotic*" OR "narcotic agent" OR "neonatal abstinence syndrome" OR "neonatal abstinence syndrome" OR "nicotin*" OR "nicotine" OR "opiate" OR "opiate*" OR "opioid" OR "opioid*" OR "opiate addiction" OR "opioids" OR "opium dependence" OR "opium" OR "opium*" OR "phencyclidine" OR "phencyclidine*" OR "smoking" OR "smoking" OR "substance abus*" OR "drug dependence treatment" OR "substance abuse" OR "substance abuse" OR "substance withdrawal" OR "substance induced" OR e"drug dependence" OR "tobacco" OR "smoking cessation" OR "tobacco dependence" OR "tobacco" OR "tobacco*" OR "dependence")) NOT TI=(("Animals" OR "veterinary" OR "rabbit" OR "rabbits" OR "animal" OR "animals" OR "mouse" OR "mice" OR "rodent" OR "rodents" OR "rat" OR "rats" OR "pig" OR "pigs" OR "porcine" OR "horse" OR "horses" OR "equine" OR "cow" OR "cows" OR "bovine" OR "goat" OR "goats" OR "sheep" OR "ovine" OR "canine" OR "dog" OR "dogs" OR "feline" OR "cat" OR "cats") NOT ("Humans" OR "Human" OR "patient" OR "patients")) NOT TI=(("Review" OR "review") NOT ("systematic review" OR "Clinical Study" OR "trial" OR "RCT")) NOT TI=(("Infant" OR "Infant" OR "Infants" OR "Child" OR "child" OR "children" OR "Adolescent" OR "adolescent" OR "adolescents" OR "adolescence") NOT ("Adult" OR "adult" OR "adults" OR "elderly")))

Cochrane

(("Psilocybine" OR "Psilocybin" OR "Psilocybins" OR "Psilocybin*" OR "Psilocibin" OR "Psilocibin*" OR "Psilocybe" OR "Psilocybe" OR "psilocin" OR "Psilocin" OR "Psilocin*" OR "omega N methyl 4 hydroxytryptamine" OR "norpsilocin" OR "norpsilocin*" OR "psychedelic agent" OR "hallucinogens" OR "hallucinogen*" OR "psychedelic" OR "psychedel*"):ti,ab,kw AND ("antiaddictive" OR "antiaddict*" OR "addict" OR "addict*" OR "addicted" OR "addiction medicine" OR "addiction" OR "addictions" OR "addicts" OR "alcohol abstinence" OR "drinking behavior" OR "alcohol withdrawal" OR "alcohol" OR "alcohol*" OR "alcoholic intoxication" OR "alcoholism" OR "alcohol induced disorders" OR "alcoholism" OR "alcohol derivative" OR "amphetamine" OR "amphetamine" OR "amphetamine*" OR "amphetamine dependence" OR "narcotic analgesic agent" OR "addiction" OR "benzodiazepine" OR "benzodiazepine*" OR "benzodiazepine" OR "benzodiazepines" OR "binge drinking" OR "cannabin*" OR "cannabis" OR "cannabis" OR "cigar" OR "cigar*" OR "cigarette" OR "cigarette*" OR "cocaine smoking" OR "cocaine" OR "cocaine" OR "cocaine*" OR "cocaine dependence" OR "crack*" OR "dexamphetamine" OR "drug overdose" OR "drug overdose" OR "alcohol" OR "ethanol*" OR "gambl*" OR "gambling" OR "heroin dependence" OR "heroin" OR "heroin*" OR "diamorphine" OR "inhalant abuse" OR "inhalant abuse" OR "ketamine" OR "ketamine*" OR "cannabis smoking" OR "marijuana smoking" OR "marijuana" OR "marijuana*" OR "methadon*" OR "methamphetamine" OR "morphine addiction" OR "morphine derivative" OR "morphine" OR "morphine" OR "morphine*" OR "narcotic" OR "narcotic*" OR "narcotic agent" OR "neonatal abstinence syndrome" OR "neonatal abstinence syndrome" OR "nicotin*" OR "nicotine" OR "opiate" OR "opiate*" OR "opioid" OR "opioid*" OR "opiate addiction" OR "opioids" OR "opium dependence" OR "opium" OR "opium*" OR "phencyclidine" OR "phencyclidine*" OR "smoking" OR "smoking" OR "substance abus*" OR "drug dependence treatment" OR "substance abuse" OR "substance abuse" OR "substance withdrawal" OR "substance induced" OR e"drug dependence" OR "tobacco" OR "smoking cessation" OR "tobacco dependence" OR "tobacco" OR "tobacco*" OR "dependence"):ti,ab,kw NOT (("Infant" OR "Infant" OR "Infants" OR "Child" OR "child" OR "children" OR "Adolescent" OR "adolescent" OR "adolescents" OR "adolescence") NOT ("Adult" OR "adult" OR "adults" OR "elderly")))

Emcare

((exp *"Psilocybine"/ OR "Psilocybin".ti,ab OR "Psilocybins".ti,ab OR "Psilocybin*".ti,ab OR "Psilocibin".ti,ab OR "Psilocibin*".ti,ab OR "Psilocybe".ti,ab OR "Psilocybe".ti,ab OR exp *"psilocin"/ OR "Psilocin".ti,ab OR "Psilocin*".ti,ab OR "omega-N-methyl-4-hydroxytryptamine".ti,ab OR "norpsilocin".ti,ab OR "norpsilocin*".ti,ab OR exp *"psychedelic agent"/ OR "hallucinogens".ti,ab OR "hallucinogen*".ti,ab OR "psychedelic".ti,ab OR "psychedel*".ti,ab) AND ("antiaddictive".ti,ab OR "antiaddict*".ti,ab OR "addict".ti,ab OR "addict*".ti,ab OR "addicted".ti,ab OR exp *"addiction medicine"/ OR "addiction".ti,ab OR "addictions".ti,ab OR "addicts".ti,ab OR exp *"alcohol abstinence"/ OR exp *"drinking behavior"/ OR "alcohol withdrawal".ti,ab OR "alcohol".ti,ab OR "alcohol*".ti,ab OR "alcoholic intoxication".ti,ab OR exp *"alcoholism"/ OR "alcohol-induced disorders".ti,ab OR "alcoholism".ti,ab OR exp *"alcohol derivative"/ OR exp *"amphetamine"/ OR "amphetamine".ti,ab OR "amphetamine*".ti,ab OR exp "amphetamine dependence"/ OR exp *"narcotic analgesic agent"/ OR exp *"addiction"/ OR "benzodiazepine".ti,ab OR "benzodiazepine*".ti,ab OR exp *"benzodiazepine"/ OR "benzodiazepines".ti,ab OR "binge drinking".ti,ab OR "cannabin*".ti,ab OR exp *"cannabis"/ OR "cannabis".ti,ab OR "cigar".ti,ab OR "cigar*".ti,ab OR "cigarette".ti,ab OR "cigarette*".ti,ab OR exp *"cocaine smoking"/ OR "cocaine"/ OR "cocaine".ti,ab OR "cocaine*".ti,ab OR exp *"cocaine dependence"/ OR "crack*".ti,ab OR exp *"dexamphetamine"/ OR exp *"drug overdose"/ OR "drug overdose".ti,ab OR exp *"alcohol"/ OR "ethanol*".ti,ab OR "gambl*".ti,ab OR exp *"gambling"/ OR exp *"heroin dependence"/ OR "heroin".ti,ab OR "heroin*".ti,ab OR exp *"diamorphine"/ OR exp *"inhalant abuse"/ OR "inhalant abuse".ti,ab OR exp *"ketamine"/ OR "ketamine*".ti,ab OR exp *"cannabis smoking"/ OR "marijuana smoking".ti,ab OR "marijuana".ti,ab OR "marijuana*".ti,ab OR "methadon*".ti,ab OR exp *"methamphetamine"/ OR exp *"morphine addiction"/ OR exp *"morphine derivative"/ OR exp *"morphine"/ OR "morphine".ti,ab OR "morphine*".ti,ab OR "narcotic".ti,ab OR "narcotic*".ti,ab OR exp *"narcotic agent"/ OR exp *"neonatal abstinence syndrome"/ OR "neonatal abstinence syndrome".ti,ab OR "nicotin*".ti,ab OR exp *"nicotine"/ OR "opiate".ti,ab OR "opiate*".ti,ab OR "opioid".ti,ab OR "opioid*".ti,ab OR exp *"opiate addiction"/ OR "opioids".ti,ab OR "opium dependence".ti,ab OR "opium".ti,ab OR "opium*".ti,ab OR "phencyclidine".ti,ab OR "phencyclidine*".ti,ab OR exp *"smoking"/ OR "smoking".ti,ab OR "substance abus*".ti,ab OR exp *"drug dependence treatment"/ OR "substance abuse".ti,ab OR exp *"substance abuse"/ OR "substance withdrawal".ti,ab OR "substance-induced".ti,ab OR eexp *"drug dependence"/ OR exp *"tobacco"/ OR exp *"smoking cessation"/ OR exp *"tobacco dependence"/ OR "tobacco".ti,ab OR "tobacco*".ti,ab OR "dependence".ti,ab) NOT ((exp "Animals"/ OR "veterinary".ti OR "rabbit".ti OR "rabbits".ti OR "animal".ti OR "animals".ti OR "mouse".ti OR "mice".ti OR "rodent".ti OR "rodents".ti OR "rat".ti OR "rats".ti OR "pig".ti OR "pigs".ti OR "porcine".ti OR "horse".ti OR "horses".ti OR "equine".ti OR "cow".ti OR "cows".ti OR "bovine".ti OR "goat".ti OR "goats".ti OR "sheep".ti OR "ovine".ti OR "canine".ti OR "dog".ti OR "dogs".ti OR "feline".ti OR "cat".ti OR "cats".ti) NOT (exp "Humans"/ OR "Humans".ti OR "Humans".ti OR "patient".ti OR "patients".ti)) NOT (("Review"/ OR "review".ti) NOT ("systematic review"/ OR exp "Clinical Study"/ OR "trial".ti OR "RCT".ti)) NOT ((exp "Infant"/ OR "Infant".ti OR "Infants".ti OR exp "Child"/ OR "child".ti OR "children".ti OR exp "Adolescent"/ OR "adolescent".ti OR "adolescents".ti OR "adolescence".ti) NOT (eexp "Adult"/ OR "adult".ti OR "adults".ti OR "elderly".ti)))

PsycINFO

Limit to Academic Journals

((TI("Psilocybine" OR "Psilocybin" OR "Psilocybins" OR "Psilocybin*" OR "Psilocibin" OR "Psilocibin*" OR "Psilocybe" OR "Psilocybe" OR "psilocin" OR "Psilocin" OR "Psilocin*" OR "omega N methyl 4 hydroxytryptamine" OR "norpsilocin" OR "norpsilocin*" OR "psychedelic agent" OR "hallucinogens" OR "hallucinogen*" OR "psychedelic" OR "psychedel*") OR SU("Psilocybine" OR "Psilocybin" OR "Psilocybins" OR "Psilocybin*" OR "Psilocibin" OR "Psilocibin*" OR "Psilocybe" OR "Psilocybe" OR "psilocin" OR "Psilocin" OR "Psilocin*" OR "omega N methyl 4 hydroxytryptamine" OR "norpsilocin" OR "norpsilocin*" OR "psychedelic agent" OR "hallucinogens" OR "hallucinogen*" OR "psychedelic" OR "psychedel*") OR MA("Psilocybine" OR "Psilocybin" OR "Psilocybins" OR "Psilocybin*" OR "Psilocibin" OR "Psilocibin*" OR "Psilocybe" OR "Psilocybe" OR "psilocin" OR "Psilocin" OR "Psilocin*" OR "omega N methyl 4 hydroxytryptamine" OR "norpsilocin" OR "norpsilocin*" OR "psychedelic agent" OR "hallucinogens" OR "hallucinogen*" OR "psychedelic" OR "psychedel*") OR AB("Psilocybine" OR "Psilocybin" OR "Psilocybins" OR "Psilocybin*" OR "Psilocibin" OR "Psilocibin*" OR "Psilocybe" OR "Psilocybe" OR "psilocin" OR "Psilocin" OR "Psilocin*" OR "omega N methyl 4 hydroxytryptamine" OR "norpsilocin" OR "norpsilocin*" OR "psychedelic agent" OR "hallucinogens" OR "hallucinogen*" OR "psychedelic" OR "psychedel*")) AND (TI("antiaddictive" OR "antiaddict*" OR "addict" OR "addict*" OR "addicted" OR "addiction medicine" OR "addiction" OR "addictions" OR "addicts" OR "alcohol abstinence" OR "drinking behavior" OR "alcohol withdrawal" OR "alcohol" OR "alcohol*" OR "alcoholic intoxication" OR "alcoholism" OR "alcohol induced disorders" OR "alcoholism" OR "alcohol derivative" OR "amphetamine" OR "amphetamine" OR "amphetamine*" OR "amphetamine dependence" OR "narcotic analgesic agent" OR "addiction" OR "benzodiazepine" OR "benzodiazepine*" OR "benzodiazepine" OR "benzodiazepines" OR "binge drinking" OR "cannabin*" OR "cannabis" OR "cannabis" OR "cigar" OR "cigar*" OR "cigarette" OR "cigarette*" OR "cocaine smoking" OR "cocaine" OR "cocaine" OR "cocaine*" OR "cocaine dependence" OR "crack*" OR "dexamphetamine" OR "drug overdose" OR "drug overdose" OR "alcohol" OR "ethanol*" OR "gambl*" OR "gambling" OR "heroin dependence" OR "heroin" OR "heroin*" OR "diamorphine" OR "inhalant abuse" OR "inhalant abuse" OR "ketamine" OR "ketamine*" OR "cannabis smoking" OR "marijuana smoking" OR "marijuana" OR "marijuana*" OR "methadon*" OR "methamphetamine" OR "morphine addiction" OR "morphine derivative" OR "morphine" OR "morphine" OR "morphine*" OR "narcotic" OR "narcotic*" OR "narcotic agent" OR "neonatal abstinence syndrome" OR "neonatal abstinence syndrome" OR "nicotin*" OR "nicotine" OR "opiate" OR "opiate*" OR "opioid" OR "opioid*" OR "opiate addiction" OR "opioids" OR "opium dependence" OR "opium" OR "opium*" OR "phencyclidine" OR "phencyclidine*" OR "smoking" OR "smoking" OR "substance abus*" OR "drug dependence treatment" OR "substance abuse" OR "substance abuse" OR "substance withdrawal" OR "substance induced" OR e"drug dependence" OR "tobacco" OR "smoking cessation" OR "tobacco dependence" OR "tobacco" OR "tobacco*" OR "dependence") OR SU("antiaddictive" OR "antiaddict*" OR "addict" OR "addict*" OR "addicted" OR "addiction medicine" OR "addiction" OR "addictions" OR "addicts" OR "alcohol abstinence" OR "drinking behavior" OR "alcohol withdrawal" OR "alcohol" OR "alcohol*" OR "alcoholic intoxication" OR "alcoholism" OR "alcohol induced disorders" OR "alcoholism" OR "alcohol derivative" OR "amphetamine" OR "amphetamine" OR "amphetamine*" OR "amphetamine dependence" OR "narcotic analgesic agent" OR "addiction" OR "benzodiazepine" OR "benzodiazepine*" OR "benzodiazepine" OR "benzodiazepines" OR "binge drinking" OR "cannabin*" OR "cannabis" OR "cannabis" OR "cigar" OR "cigar*" OR "cigarette" OR "cigarette*" OR "cocaine smoking" OR "cocaine" OR "cocaine" OR "cocaine*" OR "cocaine dependence" OR "crack*" OR "dexamphetamine" OR "drug overdose" OR "drug overdose" OR "alcohol" OR "ethanol*" OR "gambl*" OR "gambling" OR "heroin dependence" OR "heroin" OR "heroin*" OR "diamorphine" OR "inhalant abuse" OR "inhalant abuse" OR "ketamine" OR "ketamine*" OR "cannabis smoking" OR "marijuana smoking" OR "marijuana" OR "marijuana*" OR "methadon*" OR "methamphetamine" OR "morphine addiction" OR "morphine derivative" OR "morphine" OR "morphine" OR "morphine*" OR "narcotic" OR "narcotic*" OR "narcotic agent" OR "neonatal abstinence syndrome" OR "neonatal abstinence syndrome" OR "nicotin*" OR "nicotine" OR "opiate" OR "opiate*" OR "opioid" OR "opioid*" OR "opiate addiction" OR "opioids" OR "opium dependence" OR "opium" OR "opium*" OR "phencyclidine" OR "phencyclidine*" OR "smoking" OR "smoking" OR "substance abus*" OR "drug dependence treatment" OR "substance abuse" OR "substance abuse" OR "substance withdrawal" OR "substance induced" OR e"drug dependence" OR "tobacco" OR "smoking cessation" OR "tobacco dependence" OR "tobacco" OR "tobacco*" OR "dependence") OR MA("antiaddictive" OR "antiaddict*" OR "addict" OR "addict*" OR "addicted" OR "addiction medicine" OR "addiction" OR "addictions" OR "addicts" OR "alcohol abstinence" OR "drinking behavior" OR "alcohol withdrawal" OR "alcohol" OR "alcohol*" OR "alcoholic intoxication" OR "alcoholism" OR "alcohol induced disorders" OR "alcoholism" OR "alcohol derivative" OR "amphetamine" OR "amphetamine" OR "amphetamine*" OR "amphetamine dependence" OR "narcotic analgesic agent" OR "addiction" OR "benzodiazepine" OR "benzodiazepine*" OR "benzodiazepine" OR "benzodiazepines" OR "binge drinking" OR "cannabin*" OR "cannabis" OR "cannabis" OR "cigar" OR "cigar*" OR "cigarette" OR "cigarette*" OR "cocaine smoking" OR "cocaine" OR "cocaine" OR "cocaine*" OR "cocaine dependence" OR "crack*" OR "dexamphetamine" OR "drug overdose" OR "drug overdose" OR "alcohol" OR "ethanol*" OR "gambl*" OR "gambling" OR "heroin dependence" OR "heroin" OR "heroin*" OR "diamorphine" OR "inhalant abuse" OR "inhalant abuse" OR "ketamine" OR "ketamine*" OR "cannabis smoking" OR "marijuana smoking" OR "marijuana" OR "marijuana*" OR "methadon*" OR "methamphetamine" OR "morphine addiction" OR "morphine derivative" OR "morphine" OR "morphine" OR "morphine*" OR "narcotic" OR "narcotic*" OR "narcotic agent" OR "neonatal abstinence syndrome" OR "neonatal abstinence syndrome" OR "nicotin*" OR "nicotine" OR "opiate" OR "opiate*" OR "opioid" OR "opioid*" OR "opiate addiction" OR "opioids" OR "opium dependence" OR "opium" OR "opium*" OR "phencyclidine" OR "phencyclidine*" OR "smoking" OR "smoking" OR "substance abus*" OR "drug dependence treatment" OR "substance abuse" OR "substance abuse" OR "substance withdrawal" OR "substance induced" OR e"drug dependence" OR "tobacco" OR "smoking cessation" OR "tobacco dependence" OR "tobacco" OR "tobacco*" OR "dependence") OR AB("antiaddictive" OR "antiaddict*" OR "addict" OR "addict*" OR "addicted" OR "addiction medicine" OR "addiction" OR "addictions" OR "addicts" OR "alcohol abstinence" OR "drinking behavior" OR "alcohol withdrawal" OR "alcohol" OR "alcohol*" OR "alcoholic intoxication" OR "alcoholism" OR "alcohol induced disorders" OR "alcoholism" OR "alcohol derivative" OR "amphetamine" OR "amphetamine" OR "amphetamine*" OR "amphetamine dependence" OR "narcotic analgesic agent" OR "addiction" OR "benzodiazepine" OR "benzodiazepine*" OR "benzodiazepine" OR "benzodiazepines" OR "binge drinking" OR "cannabin*" OR "cannabis" OR "cannabis" OR "cigar" OR "cigar*" OR "cigarette" OR "cigarette*" OR "cocaine smoking" OR "cocaine" OR "cocaine" OR "cocaine*" OR "cocaine dependence" OR "crack*" OR "dexamphetamine" OR "drug overdose" OR "drug overdose" OR "alcohol" OR "ethanol*" OR "gambl*" OR "gambling" OR "heroin dependence" OR "heroin" OR "heroin*" OR "diamorphine" OR "inhalant abuse" OR "inhalant abuse" OR "ketamine" OR "ketamine*" OR "cannabis smoking" OR "marijuana smoking" OR "marijuana" OR "marijuana*" OR "methadon*" OR "methamphetamine" OR "morphine addiction" OR "morphine derivative" OR "morphine" OR "morphine" OR "morphine*" OR "narcotic" OR "narcotic*" OR "narcotic agent" OR "neonatal abstinence syndrome" OR "neonatal abstinence syndrome" OR "nicotin*" OR "nicotine" OR "opiate" OR "opiate*" OR "opioid" OR "opioid*" OR "opiate addiction" OR "opioids" OR "opium dependence" OR "opium" OR "opium*" OR "phencyclidine" OR "phencyclidine*" OR "smoking" OR "smoking" OR "substance abus*" OR "drug dependence treatment" OR "substance abuse" OR "substance abuse" OR "substance withdrawal" OR "substance induced" OR e"drug dependence" OR "tobacco" OR "smoking cessation" OR "tobacco dependence" OR "tobacco" OR "tobacco*" OR "dependence")) NOT TI(("Animals" OR "veterinary" OR "rabbit" OR "rabbits" OR "animal" OR "animals" OR "mouse" OR "mice" OR "rodent" OR "rodents" OR "rat" OR "rats" OR "pig" OR "pigs" OR "porcine" OR "horse" OR "horses" OR "equine" OR "cow" OR "cows" OR "bovine" OR "goat" OR "goats" OR "sheep" OR "ovine" OR "canine" OR "dog" OR "dogs" OR "feline" OR "cat" OR "cats") NOT ("Humans" OR "Human" OR "patient" OR "patients")) NOT TI(("Review" OR "review") NOT ("systematic review" OR "Clinical Study" OR "trial" OR "RCT")) NOT TI(("Infant" OR "Infant" OR "Infants" OR "Child" OR "child" OR "children" OR "Adolescent" OR "adolescent" OR "adolescents" OR "adolescence") NOT ("Adult" OR "adult" OR "adults" OR "elderly")))

Academic Search Premier

Limit to Academic Journals

((TI("Psilocybine" OR "Psilocybin" OR "Psilocybins" OR "Psilocybin*" OR "Psilocibin" OR "Psilocibin*" OR "Psilocybe" OR "Psilocybe" OR "psilocin" OR "Psilocin" OR "Psilocin*" OR "omega N methyl 4 hydroxytryptamine" OR "norpsilocin" OR "norpsilocin*" OR "psychedelic agent" OR "hallucinogens" OR "hallucinogen*" OR "psychedelic" OR "psychedel*") OR SU("Psilocybine" OR "Psilocybin" OR "Psilocybins" OR "Psilocybin*" OR "Psilocibin" OR "Psilocibin*" OR "Psilocybe" OR "Psilocybe" OR "psilocin" OR "Psilocin" OR "Psilocin*" OR "omega N methyl 4 hydroxytryptamine" OR "norpsilocin" OR "norpsilocin*" OR "psychedelic agent" OR "hallucinogens" OR "hallucinogen*" OR "psychedelic" OR "psychedel*") OR KW("Psilocybine" OR "Psilocybin" OR "Psilocybins" OR "Psilocybin*" OR "Psilocibin" OR "Psilocibin*" OR "Psilocybe" OR "Psilocybe" OR "psilocin" OR "Psilocin" OR "Psilocin*" OR "omega N methyl 4 hydroxytryptamine" OR "norpsilocin" OR "norpsilocin*" OR "psychedelic agent" OR "hallucinogens" OR "hallucinogen*" OR "psychedelic" OR "psychedel*")) AND (TI("antiaddictive" OR "antiaddict*" OR "addict" OR "addict*" OR "addicted" OR "addiction medicine" OR "addiction" OR "addictions" OR "addicts" OR "alcohol abstinence" OR "drinking behavior" OR "alcohol withdrawal" OR "alcohol" OR "alcohol*" OR "alcoholic intoxication" OR "alcoholism" OR "alcohol induced disorders" OR "alcoholism" OR "alcohol derivative" OR "amphetamine" OR "amphetamine" OR "amphetamine*" OR "amphetamine dependence" OR "narcotic analgesic agent" OR "addiction" OR "benzodiazepine" OR "benzodiazepine*" OR "benzodiazepine" OR "benzodiazepines" OR "binge drinking" OR "cannabin*" OR "cannabis" OR "cannabis" OR "cigar" OR "cigar*" OR "cigarette" OR "cigarette*" OR "cocaine smoking" OR "cocaine" OR "cocaine" OR "cocaine*" OR "cocaine dependence" OR "crack*" OR "dexamphetamine" OR "drug overdose" OR "drug overdose" OR "alcohol" OR "ethanol*" OR "gambl*" OR "gambling" OR "heroin dependence" OR "heroin" OR "heroin*" OR "diamorphine" OR "inhalant abuse" OR "inhalant abuse" OR "ketamine" OR "ketamine*" OR "cannabis smoking" OR "marijuana smoking" OR "marijuana" OR "marijuana*" OR "methadon*" OR "methamphetamine" OR "morphine addiction" OR "morphine derivative" OR "morphine" OR "morphine" OR "morphine*" OR "narcotic" OR "narcotic*" OR "narcotic agent" OR "neonatal abstinence syndrome" OR "neonatal abstinence syndrome" OR "nicotin*" OR "nicotine" OR "opiate" OR "opiate*" OR "opioid" OR "opioid*" OR "opiate addiction" OR "opioids" OR "opium dependence" OR "opium" OR "opium*" OR "phencyclidine" OR "phencyclidine*" OR "smoking" OR "smoking" OR "substance abus*" OR "drug dependence treatment" OR "substance abuse" OR "substance abuse" OR "substance withdrawal" OR "substance induced" OR e"drug dependence" OR "tobacco" OR "smoking cessation" OR "tobacco dependence" OR "tobacco" OR "tobacco*" OR "dependence") OR SU("antiaddictive" OR "antiaddict*" OR "addict" OR "addict*" OR "addicted" OR "addiction medicine" OR "addiction" OR "addictions" OR "addicts" OR "alcohol abstinence" OR "drinking behavior" OR "alcohol withdrawal" OR "alcohol" OR "alcohol*" OR "alcoholic intoxication" OR "alcoholism" OR "alcohol induced disorders" OR "alcoholism" OR "alcohol derivative" OR "amphetamine" OR "amphetamine" OR "amphetamine*" OR "amphetamine dependence" OR "narcotic analgesic agent" OR "addiction" OR "benzodiazepine" OR "benzodiazepine*" OR "benzodiazepine" OR "benzodiazepines" OR "binge drinking" OR "cannabin*" OR "cannabis" OR "cannabis" OR "cigar" OR "cigar*" OR "cigarette" OR "cigarette*" OR "cocaine smoking" OR "cocaine" OR "cocaine" OR "cocaine*" OR "cocaine dependence" OR "crack*" OR "dexamphetamine" OR "drug overdose" OR "drug overdose" OR "alcohol" OR "ethanol*" OR "gambl*" OR "gambling" OR "heroin dependence" OR "heroin" OR "heroin*" OR "diamorphine" OR "inhalant abuse" OR "inhalant abuse" OR "ketamine" OR "ketamine*" OR "cannabis smoking" OR "marijuana smoking" OR "marijuana" OR "marijuana*" OR "methadon*" OR "methamphetamine" OR "morphine addiction" OR "morphine derivative" OR "morphine" OR "morphine" OR "morphine*" OR "narcotic" OR "narcotic*" OR "narcotic agent" OR "neonatal abstinence syndrome" OR "neonatal abstinence syndrome" OR "nicotin*" OR "nicotine" OR "opiate" OR "opiate*" OR "opioid" OR "opioid*" OR "opiate addiction" OR "opioids" OR "opium dependence" OR "opium" OR "opium*" OR "phencyclidine" OR "phencyclidine*" OR "smoking" OR "smoking" OR "substance abus*" OR "drug dependence treatment" OR "substance abuse" OR "substance abuse" OR "substance withdrawal" OR "substance induced" OR e"drug dependence" OR "tobacco" OR "smoking cessation" OR "tobacco dependence" OR "tobacco" OR "tobacco*" OR "dependence") OR KW("antiaddictive" OR "antiaddict*" OR "addict" OR "addict*" OR "addicted" OR "addiction medicine" OR "addiction" OR "addictions" OR "addicts" OR "alcohol abstinence" OR "drinking behavior" OR "alcohol withdrawal" OR "alcohol" OR "alcohol*" OR "alcoholic intoxication" OR "alcoholism" OR "alcohol induced disorders" OR "alcoholism" OR "alcohol derivative" OR "amphetamine" OR "amphetamine" OR "amphetamine*" OR "amphetamine dependence" OR "narcotic analgesic agent" OR "addiction" OR "benzodiazepine" OR "benzodiazepine*" OR "benzodiazepine" OR "benzodiazepines" OR "binge drinking" OR "cannabin*" OR "cannabis" OR "cannabis" OR "cigar" OR "cigar*" OR "cigarette" OR "cigarette*" OR "cocaine smoking" OR "cocaine" OR "cocaine" OR "cocaine*" OR "cocaine dependence" OR "crack*" OR "dexamphetamine" OR "drug overdose" OR "drug overdose" OR "alcohol" OR "ethanol*" OR "gambl*" OR "gambling" OR "heroin dependence" OR "heroin" OR "heroin*" OR "diamorphine" OR "inhalant abuse" OR "inhalant abuse" OR "ketamine" OR "ketamine*" OR "cannabis smoking" OR "marijuana smoking" OR "marijuana" OR "marijuana*" OR "methadon*" OR "methamphetamine" OR "morphine addiction" OR "morphine derivative" OR "morphine" OR "morphine" OR "morphine*" OR "narcotic" OR "narcotic*" OR "narcotic agent" OR "neonatal abstinence syndrome" OR "neonatal abstinence syndrome" OR "nicotin*" OR "nicotine" OR "opiate" OR "opiate*" OR "opioid" OR "opioid*" OR "opiate addiction" OR "opioids" OR "opium dependence" OR "opium" OR "opium*" OR "phencyclidine" OR "phencyclidine*" OR "smoking" OR "smoking" OR "substance abus*" OR "drug dependence treatment" OR "substance abuse" OR "substance abuse" OR "substance withdrawal" OR "substance induced" OR e"drug dependence" OR "tobacco" OR "smoking cessation" OR "tobacco dependence" OR "tobacco" OR "tobacco*" OR "dependence")) NOT TI(("Animals" OR "veterinary" OR "rabbit" OR "rabbits" OR "animal" OR "animals" OR "mouse" OR "mice" OR "rodent" OR "rodents" OR "rat" OR "rats" OR "pig" OR "pigs" OR "porcine" OR "horse" OR "horses" OR "equine" OR "cow" OR "cows" OR "bovine" OR "goat" OR "goats" OR "sheep" OR "ovine" OR "canine" OR "dog" OR "dogs" OR "feline" OR "cat" OR "cats") NOT ("Humans" OR "Human" OR "patient" OR "patients")) NOT TI(("Review" OR "review") NOT ("systematic review" OR "Clinical Study" OR "trial" OR "RCT")) NOT TI(("Infant" OR "Infant" OR "Infants" OR "Child" OR "child" OR "children" OR "Adolescent" OR "adolescent" OR "adolescents" OR "adolescence") NOT ("Adult" OR "adult" OR "adults" OR "elderly")))

**OR**

(TI("Psilocybine" OR "Psilocybin" OR "Psilocybins" OR "Psilocybin*" OR "Psilocibin" OR "Psilocibin*" OR "Psilocybe" OR "Psilocybe" OR "psilocin" OR "Psilocin" OR "Psilocin*" OR "omega N methyl 4 hydroxytryptamine" OR "norpsilocin" OR "norpsilocin*" OR "psychedelic agent" OR "hallucinogens" OR "hallucinogen*" OR "psychedelic" OR "psychedel*") AND AB("antiaddictive" OR "antiaddict*" OR "addict" OR "addict*" OR "addicted" OR "addiction medicine" OR "addiction" OR "addictions" OR "addicts" OR "alcohol abstinence" OR "drinking behavior" OR "alcohol withdrawal" OR "alcohol" OR "alcohol*" OR "alcoholic intoxication" OR "alcoholism" OR "alcohol induced disorders" OR "alcoholism" OR "alcohol derivative" OR "amphetamine" OR "amphetamine" OR "amphetamine*" OR "amphetamine dependence" OR "narcotic analgesic agent" OR "addiction" OR "benzodiazepine" OR "benzodiazepine*" OR "benzodiazepine" OR "benzodiazepines" OR "binge drinking" OR "cannabin*" OR "cannabis" OR "cannabis" OR "cigar" OR "cigar*" OR "cigarette" OR "cigarette*" OR "cocaine smoking" OR "cocaine" OR "cocaine" OR "cocaine*" OR "cocaine dependence" OR "crack*" OR "dexamphetamine" OR "drug overdose" OR "drug overdose" OR "alcohol" OR "ethanol*" OR "gambl*" OR "gambling" OR "heroin dependence" OR "heroin" OR "heroin*" OR "diamorphine" OR "inhalant abuse" OR "inhalant abuse" OR "ketamine" OR "ketamine*" OR "cannabis smoking" OR "marijuana smoking" OR "marijuana" OR "marijuana*" OR "methadon*" OR "methamphetamine" OR "morphine addiction" OR "morphine derivative" OR "morphine" OR "morphine" OR "morphine*" OR "narcotic" OR "narcotic*" OR "narcotic agent" OR "neonatal abstinence syndrome" OR "neonatal abstinence syndrome" OR "nicotin*" OR "nicotine" OR "opiate" OR "opiate*" OR "opioid" OR "opioid*" OR "opiate addiction" OR "opioids" OR "opium dependence" OR "opium" OR "opium*" OR "phencyclidine" OR "phencyclidine*" OR "smoking" OR "smoking" OR "substance abus*" OR "drug dependence treatment" OR "substance abuse" OR "substance abuse" OR "substance withdrawal" OR "substance induced" OR e"drug dependence" OR "tobacco" OR "smoking cessation" OR "tobacco dependence" OR "tobacco" OR "tobacco*" OR "dependence") NOT TI(("Animals" OR "veterinary" OR "rabbit" OR "rabbits" OR "animal" OR "animals" OR "mouse" OR "mice" OR "rodent" OR "rodents" OR "rat" OR "rats" OR "pig" OR "pigs" OR "porcine" OR "horse" OR "horses" OR "equine" OR "cow" OR "cows" OR "bovine" OR "goat" OR "goats" OR "sheep" OR "ovine" OR "canine" OR "dog" OR "dogs" OR "feline" OR "cat" OR "cats") NOT ("Humans" OR "Human" OR "patient" OR "patients")) NOT TI(("Review" OR "review") NOT ("systematic review" OR "Clinical Study" OR "trial" OR "RCT")) NOT TI(("Infant" OR "Infant" OR "Infants" OR "Child" OR "child" OR "children" OR "Adolescent" OR "adolescent" OR "adolescents" OR "adolescence") NOT ("Adult" OR "adult" OR "adults" OR "elderly")))

**OR**

(AB("Psilocybine" OR "Psilocybin" OR "Psilocybins" OR "Psilocybin*" OR "Psilocibin" OR "Psilocibin*" OR "Psilocybe" OR "Psilocybe" OR "psilocin" OR "Psilocin" OR "Psilocin*" OR "omega N methyl 4 hydroxytryptamine" OR "norpsilocin" OR "norpsilocin*" OR "psychedelic agent" OR "hallucinogens" OR "hallucinogen*" OR "psychedelic" OR "psychedel*") AND TI("antiaddictive" OR "antiaddict*" OR "addict" OR "addict*" OR "addicted" OR "addiction medicine" OR "addiction" OR "addictions" OR "addicts" OR "alcohol abstinence" OR "drinking behavior" OR "alcohol withdrawal" OR "alcohol" OR "alcohol*" OR "alcoholic intoxication" OR "alcoholism" OR "alcohol induced disorders" OR "alcoholism" OR "alcohol derivative" OR "amphetamine" OR "amphetamine" OR "amphetamine*" OR "amphetamine dependence" OR "narcotic analgesic agent" OR "addiction" OR "benzodiazepine" OR "benzodiazepine*" OR "benzodiazepine" OR "benzodiazepines" OR "binge drinking" OR "cannabin*" OR "cannabis" OR "cannabis" OR "cigar" OR "cigar*" OR "cigarette" OR "cigarette*" OR "cocaine smoking" OR "cocaine" OR "cocaine" OR "cocaine*" OR "cocaine dependence" OR "crack*" OR "dexamphetamine" OR "drug overdose" OR "drug overdose" OR "alcohol" OR "ethanol*" OR "gambl*" OR "gambling" OR "heroin dependence" OR "heroin" OR "heroin*" OR "diamorphine" OR "inhalant abuse" OR "inhalant abuse" OR "ketamine" OR "ketamine*" OR "cannabis smoking" OR "marijuana smoking" OR "marijuana" OR "marijuana*" OR "methadon*" OR "methamphetamine" OR "morphine addiction" OR "morphine derivative" OR "morphine" OR "morphine" OR "morphine*" OR "narcotic" OR "narcotic*" OR "narcotic agent" OR "neonatal abstinence syndrome" OR "neonatal abstinence syndrome" OR "nicotin*" OR "nicotine" OR "opiate" OR "opiate*" OR "opioid" OR "opioid*" OR "opiate addiction" OR "opioids" OR "opium dependence" OR "opium" OR "opium*" OR "phencyclidine" OR "phencyclidine*" OR "smoking" OR "smoking" OR "substance abus*" OR "drug dependence treatment" OR "substance abuse" OR "substance abuse" OR "substance withdrawal" OR "substance induced" OR e"drug dependence" OR "tobacco" OR "smoking cessation" OR "tobacco dependence" OR "tobacco" OR "tobacco*" OR "dependence") NOT TI(("Animals" OR "veterinary" OR "rabbit" OR "rabbits" OR "animal" OR "animals" OR "mouse" OR "mice" OR "rodent" OR "rodents" OR "rat" OR "rats" OR "pig" OR "pigs" OR "porcine" OR "horse" OR "horses" OR "equine" OR "cow" OR "cows" OR "bovine" OR "goat" OR "goats" OR "sheep" OR "ovine" OR "canine" OR "dog" OR "dogs" OR "feline" OR "cat" OR "cats") NOT ("Humans" OR "Human" OR "patient" OR "patients")) NOT TI(("Review" OR "review") NOT ("systematic review" OR "Clinical Study" OR "trial" OR "RCT")) NOT TI(("Infant" OR "Infant" OR "Infants" OR "Child" OR "child" OR "children" OR "Adolescent" OR "adolescent" OR "adolescents" OR "adolescence") NOT ("Adult" OR "adult" OR "adults" OR "elderly")))
